# Novel PD-L1- and collagen-expressing patient-derived cell line of undifferentiated pleomorphic sarcoma (JBT19) as a model for cancer immunotherapy

**DOI:** 10.1038/s41598-023-46305-7

**Published:** 2023-11-04

**Authors:** Pavla Taborska, Pavol Lukac, Dmitry Stakheev, Lenka Rajsiglova, Katerina Kalkusova, Karolina Strnadova, Lukas Lacina, Barbora Dvorankova, Jiri Novotny, Michal Kolar, Milena Vrana, Hana Cechova, Sarka Ransdorfova, Marie Valerianova, Karel Smetana, Luca Vannucci, Daniel Smrz

**Affiliations:** 1 Department of Immunology, Second Faculty of Medicine, Charles University, and Motol University Hospital, V Uvalu 84, 150 06 Praha 5, Prague, Czech Republic; 2 Institute of Anatomy, First Faculty of Medicine, Charles University, Prague, Czech Republic; 3 First Faculty of Medicine, BIOCEV, Charles University, Vestec, Czech Republic; 4 Laboratory of Immunotherapy, Institute of Microbiology of the Czech Academy of Sciences, Prague, Czech Republic; 5 Laboratory of Genomics and Bioinformatics, Institute of Molecular Genetics of the Czech Academy of Sciences, Prague, Czech Republic; 6 HLA Department, Institute of Hematology and Blood Transfusion, Prague, Czech Republic; 7 Department of Cytogenetics, Institute of Hematology and Blood Transfusion, Prague, Czech Republic; 8 Department of Cell Biology, Faculty of Science, Charles University, Prague, Czech Republic; 9 Department of Dermatovenerology, First Faculty of Medicine, Charles University, and General University Hospital, Prague, Czech Republic

**Keywords:** Cancer microenvironment, Cancer therapy, Sarcoma, Cancer, Immunology, Immune evasion, Immunotherapy, Translational immunology, Tumour immunology

## Abstract

Soft tissue sarcomas are aggressive mesenchymal-origin malignancies. Undifferentiated pleomorphic sarcoma (UPS) belongs to the aggressive, high-grade, and least characterized sarcoma subtype, affecting multiple tissues and metastasizing to many organs. The treatment of localized UPS includes surgery in combination with radiation therapy. Metastatic forms are treated with chemotherapy. Immunotherapy is a promising treatment modality for many cancers. However, the development of immunotherapy for UPS is limited due to its heterogeneity, antigenic landscape variation, lower infiltration with immune cells, and a limited number of established patient-derived UPS cell lines for preclinical research. In this study, we established and characterized a novel patient-derived UPS cell line, JBT19. The JBT19 cells express PD-L1 and collagen, a ligand of the immune checkpoint molecule LAIR-1. JBT19 cells can form spheroids in vitro and solid tumors in immunodeficient nude mice. We found JBT19 cells induce expansion of JBT19-reactive autologous and allogeneic NK, T, and NKT-like cells, and the reactivity of the expanded cells was associated with cytotoxic impact on JBT19 cells. The PD-1 and LAIR-1 ligand-expressing JBT19 cells show ex vivo immunogenicity and effective in vivo xenoengraftment properties that can offer a unique resource in the preclinical research developing novel immunotherapeutic interventions in the treatment of UPS.

## Introduction

Soft tissue sarcomas (STSs) are rare tumors accounting for less than 1% of diagnosed malignancies^1^. STSs comprise heterogeneous cell populations with mesenchymal features and are classified into more than 70 histological subtypes^2^. Undifferentiated pleomorphic sarcoma (UPS) is a soft tissue sarcoma subtype formerly known as malignant fibrous histiocytoma^3^. Currently, UPS is incorporated under malignant tumors of uncertain differentiation as determined by the absence of specific lines of differentiation^4^. It is an STS subtype supposed to be of fibrohistiocytic or fibroblastic lineage with various cellular backgrounds, mutational signatures, and altered signaling pathways^5^. UPS remains one of the three most frequent histotypes of STS^1^ and one with the worst prognosis^6^. It is often diagnosed as high-grade tumors with aggressive clinical behavior and with lesions enlarging painlessly, metastasizing in approximately one-third of cases, and with a 60% overall survival rate^7^. Nonmetastatic forms are treated by surgery, often followed by radiation therapy, and metastatic forms are treated by chemotherapy, but the 5-year survival rate of this form of tumor is only 16%^5^. Although UPS belongs to the three most frequent STSs, limited knowledge is still available of this STS subtype^8,9^.

Immunotherapy has been a progressively developing pillar of cancer treatment in recent years. For some types of cancers, immunotherapy has already shown impressive results, notably where the traditional treatment options had already failed (i.e., melanoma, lung cancer)^10^. Immune checkpoint inhibitors (ICIs) are highly promising treatment options for many cancers, and in some of them, ICIs are already approved for first-line therapy^11,12^. The second promising immunotherapeutic treatment option is adoptive cellular immunotherapy (ACI), which is a personalized therapy based on ex vivo-modified/expanded immune cells, which are transferred to patients to either induce a cancer-targeted immune response (active adoptive cellular immunotherapy)^13^ or to target and eliminate cancer cells directly (passive adoptive cellular immunotherapy) ^14^. However, immunotherapeutic options for many STS patients are limited due to multiple mechanisms of tumor immune resistance, large heterogeneity of STSs, and poor understanding of the STS immune microenvironment^15^. UPS is one of few STS subtypes where immunotherapy showed encouraging results, namely with anti-PD-1/PD-L-1 immunotherapy^16^. However, preclinical research on this STS subtype is limited due to a limited number of available patient-derived UPS cell lines. Despite UPS belonging to the three most frequent STSs, accounting for 10% of all STSs^5^, there are only 43 patient-derived UPS cell lines out of the total 844 patient-derived sarcoma cell lines^17^. Out of these 43 UPS cell lines, there are only 5 of them available from public repositories^17^. To our knowledge, in addition, none of these cell lines were reported to simultaneously express PD-L1 and collagen, respectively, ligands of two known immune checkpoint molecules, PD-1 and the leukocyte-associated immunoglobulin-like receptor 1 (LAIR-1). LAIR-1 is currently considered to play a role in the resistance of solid tumors to immunotherapy^18–20^, including immunotherapy based on ICIs and ACI^21^.

In this study, we describe a newly established patient-derived UPS cell line named JBT19, which simultaneously expresses PD-L1, the ligand for PD-1, and collagen, the ligand for LAIR-1. We also demonstrate JBT19's potential for applications in anticancer preclinical research, including research related to the immunotherapy of UPS.

## Results

### JBT19 cell establishment

**Figure 1:**
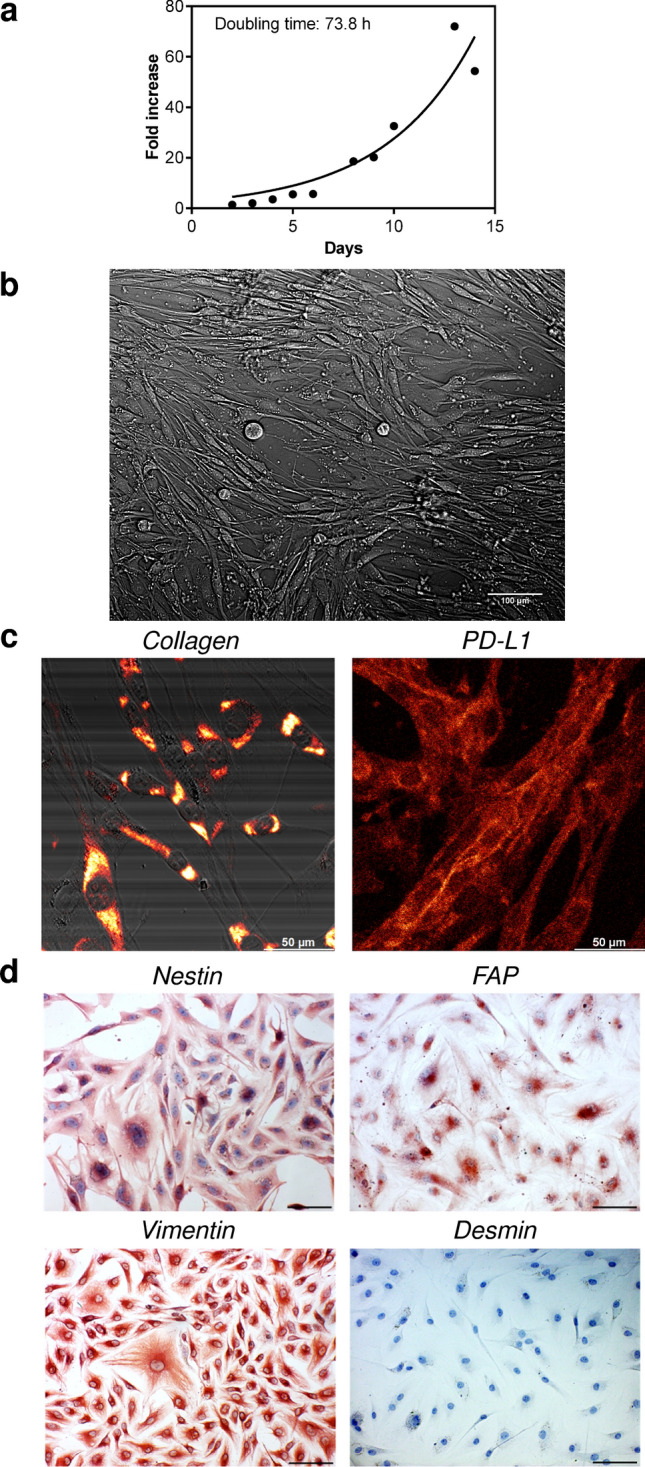
Characterization of JBT19 cells. (**a**) JBT19 growth curve. (**b**) Brightfield microscopy of adherent JBT19 cells. (**c**) Confocal microscopy of JBT19 cells intracellularly stained with collagen- or PD-L1-specific antibody. (**d**) Immunohistochemistry of JBT19 cells stained with nestin- (top left panel), FAP- (top right panel), vimentin- (bottom left panel), or desmin- (bottom right panel) specific antibody. In (**b**) and (** d**), the bars represent 100 μm. In (**c**), the bar represents 50 μm.

The JBT19 cells were established from the solid UPS primary tumor of a 65-year-old male diagnosed with the disease in August 2019. The tumor was surgically removed prior to any other therapeutic interventions. The cell line was established in adherent monolayer (two-dimensional, 2D) culture using a combination of tumor fragmentation-dissociation techniques previously described^22–25^. The JBT19 cells went through over 30 passages and were cultured for more than 30 months. They were frozen and cryopreserved in nitrogen, easily reconstituted in 2D culture, and showed a 3-day doubling time (Fig. 1a). Their morphology was prevalently fibroblastoid, and the cells tended to line up parallel-wise then assuming vortex or nest-like orientation when more confluent (Fig. 1b). The cryopreserved aliquots of the established JBT19 cells were deposited in the European Collection of Authenticated Cell Cultures (Accession number given by the International Depository Authority: 22113001, date: Dec 15, 2022). The QC testing at the Depository confirmed the cell viability of over 90% after the reconstitution, and the cells were proved mycoplasma-free.

### JBT19 phenotypic characterization

**Figure 2:**
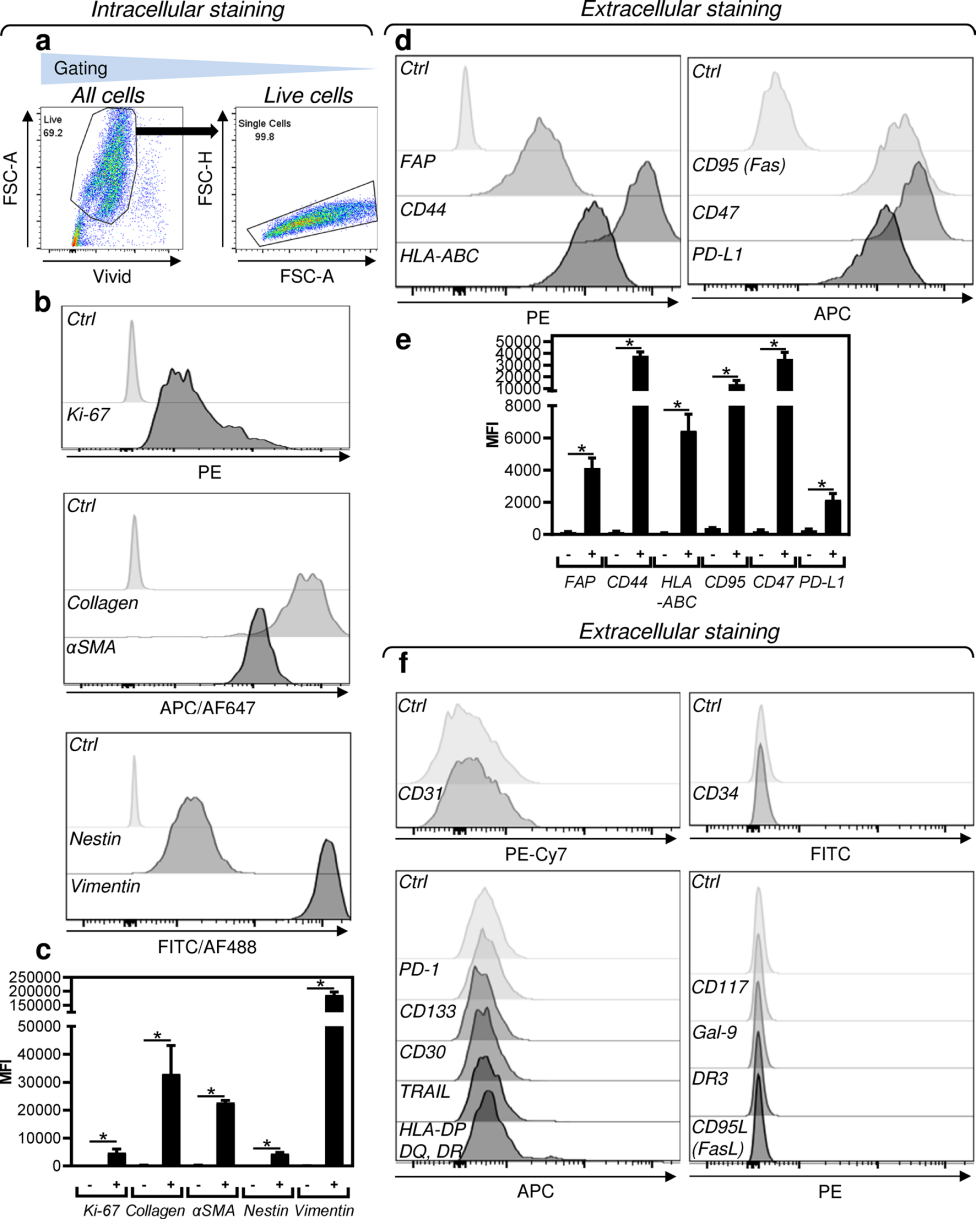
JBT19 phenotypic characterization. (**a**) The gating strategy of flow cytometry data. (**b**) Intracellular staining with Ki-67, collagen-, αSMA-, nestin-, or vimentin-specific antibody. (**c**) The evaluation of the mean fluorescence intensity (MFI) staining in **b**. (**d**) Extracellular staining with FAP-, CD44-, HLA-ABC, CD95(Fas)-, CD47-, or PD-L1-specific antibody. (**e**) The evaluation of the mean fluorescence intensity (MFI) staining in **d**. (**f**) Extracellular staining with CD31-, PD-1-, CD133-, CD30-, TRAIL-, HLA-DP, DQ, DR-, CD34-, CD117-, Gal-9-, DR3-, or CD95L(FasL)-specific antibody. In (**b**), (**d**), and (**f**), representative histograms are shown, and Ctrl for individual fluorochromes means staining with the vehicle alone. In (**c**) and (**e**), mean + SEM and statistical significances of differences between the group of unstained and stained samples are indicated (*p < 0.05; *n* = 6 (FAP, CD44), *n* = 5 (Ki-67, Collagen, αSMA, nestin, CD47, PD-L1), *n* = 4 (vimentin, HLA-ABC, CD95); paired two-tailed Student’s t-test).

The phenotypic characterization was performed using microscopy, immunohistochemistry, and flow cytometry. The analyses by confocal microscopy revealed that JBT19 cells expressed collagen and PD-L1 (Fig. 1c). Immunohistochemistry revealed the expression of nestin, fibroblast activation protein-α (FAP), and vimentin, but not desmin (Fig. 1d). An extended phenotypic analysis by intracellular staining and flow cytometry confirmed the expression of collagen, nestin, and vimentin (Fig. 2a–c). It also revealed the expression of α-smooth muscle actin (αSMA) and Ki-67 (Fig. 2a–c). The extracellular staining analysis confirmed the expression of PD-L1 and FAP and further revealed the expression of CD95 (Fas), CD44, CD47, and MHC-I (HLA-A, B, C) molecules (Fig. 2d and e). On the other hand, the JBT19 cells were found negative for the surface expression of PD-1, Galectin-9 (Gal-9), CD34, CD117, CD133, TRAIL, CD95L (FasL), DR3, CD30, CD31, and MHC-II (HLA-DP, DQ, DR) molecules (Fig. 2f).

### STR, HLA genotype, and karyotype of JBT19 cells

**Figure 3:**
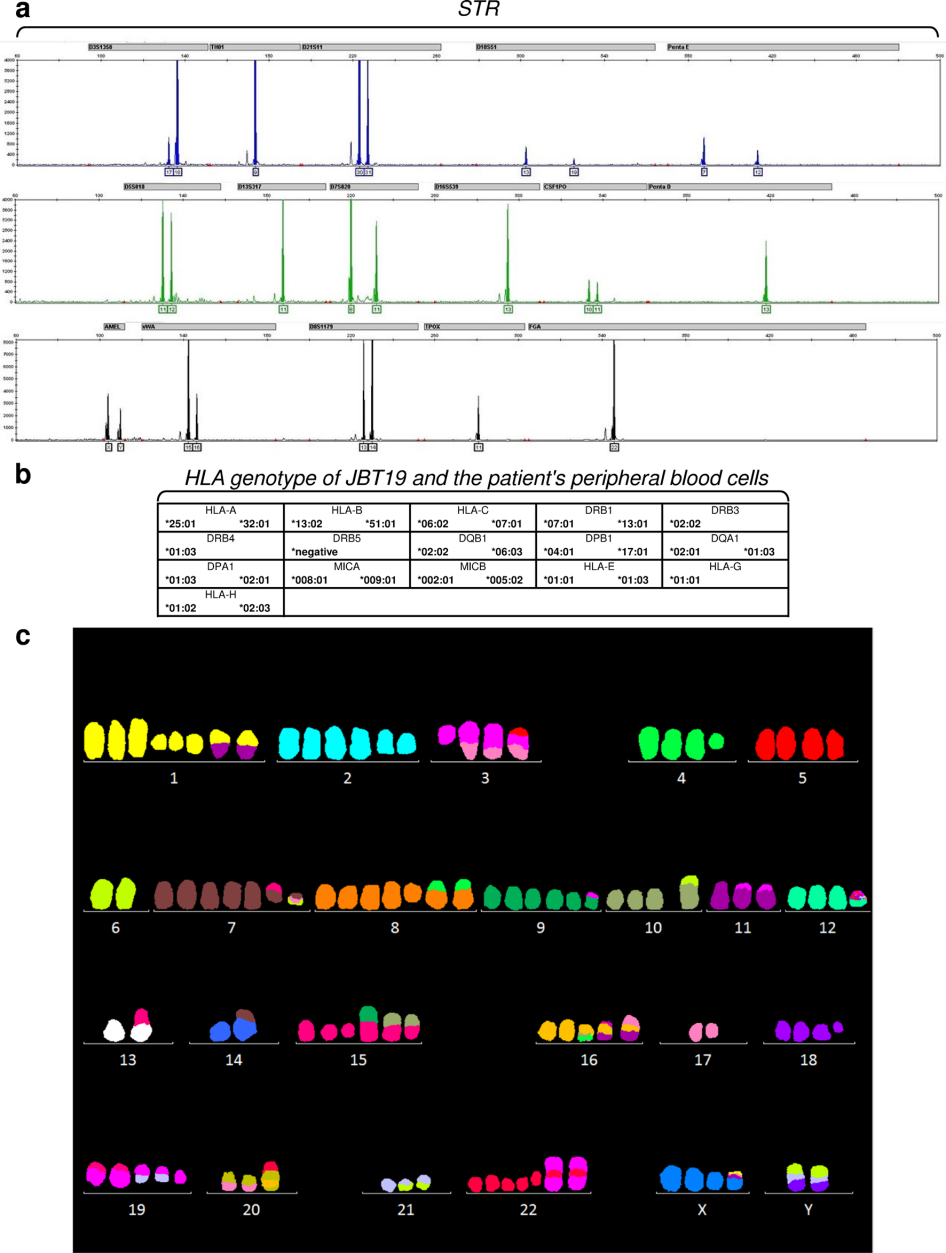
Short tandem repeats, HLA genotype, and karyotype characterization of JBT19 cells. (**a**) JBT19 short tandem repeats (STR). (**b**) HLA genotype of JBT19 cells and peripheral blood cells of the patient. (**c**) Karyotype analysis by probe 24XCyte mFISH Probe (MetaSystems). One metaphase representing a composite karyotype (in this cell some abnormalities are absent, some non-clonal abnormalities are present).

The established JBT19 cells were validated by 15 STRs loci located on 13 chromosomes and 1 sex-specific locus. For some loci, the data showed that it is not possible to determine with certainty whether it is a true allele or a PCR reaction artifact, the so-called stutter peak. The stutter product is usually about 5–15% of the area of the right allele of the given locus. The imbalance of peaks in heterozygotes was observed. It is a common phenomenon in the amplification of cell lines. These alleles represent at locus D3S1358 10%, D21S11 40%, D18S51 40%, Penta E 55%, D5S818 80%, D7S820 57%, vWA 34% and D8S1179 43% area of the right allele. Loss of heterozygosity occurred in THO1 (11p15.5), D13S317 (13q22-q31), Penta D (21q), and TPOX (2p24-2pter) loci (Fig. 3a). HLA genotype of classical and non-classical loci is specified in Fig. 3b. No allele disbalance was detected. We next examined the HLA genotype of the patient's peripheral blood cells. The analysis revealed that the patient's peripheral blood cell HLA genotype was identical to the JBT19 HLA genotype shown in Fig. 3b, thus confirming the patient's origin of the JBT19 cells.

UPS is highly heterogeneous, with multiple defects in the karyotype of the transformed cells^26^. Therefore, we next investigated the karyotype of the established JBT19 cells. Using multicolor fluorescence in situ hybridization (mFISH), we revealed that JBT19 cells are a near-tetraploid cell line (92 ±) with multiple numerical and structural changes of chromosomes (Fig. 3c). We found aberrated XY sex chromosomes and numerous numerical and structural aberrations in all 16 analyzed metaphases. The line has complex rearrangements of karyotype (≥ 3 changes, while at least two changes must be structural or not only numerical). The changes concern (practically) all chromosomes. We found frequent non-random losses of chromosomes derived from near-tetraploid (4n ±): 6, 11, 13, 14, 17 and 21. Conversely, frequent non-random gains of chromosomes derived from near-tetraploid (4n ±): 1, 2, 7, 8, 9, 15, 16, 19, 22 and X. We demonstrated only one balanced translocation t(6;10)(p12;p12). Most derived and duplicated chromosomes arose from unbalanced rearrangements. The findings were described according to the international system for human cytogenomic nomenclature (ISCN) 2020^27^, and the description is shown in Supplemental Fig. S1a. The karyotype entry is recorded as composite karyotype [cp16]—composed of karyotypes of 16 cells with chromosome number 98–109 and including all clonally occurring changes (clonal change = every change that is represented in at least two cells). Losses of various chromosomes and non-clonal aberrations (the change occurred only once) were also frequently present in the metaphases. The data from the karyotype analysis showed that JBT19 cells are indeed genetically highly transformed cells, which was consistent with previous findings in other cell lines^28,29^.

We next investigated whether the chromosomal rearrangements impacted the genes whose alterations are often observed in soft tissue sarcomas and are associated with cancer development. Among these genes are *CDKN2A* gene, located at the chromosomal region 9p21^30^, and the *RB1* gene, located at the chromosomal region 13q14^31^. Using I-FISH with the CDKN2A probe, we confirmed the loss of the *CDKN2A* gene in 174/200 (87%) interphase nuclei (Supplemental Fig. S1b). On the other hand, I-FISH with the RB1 probe confirmed no deletion of the *RB1* gene in all (0/200) of the tested interphase nuclei.

### Transcriptome profiling of JBT19 cells

**Figure 4:**
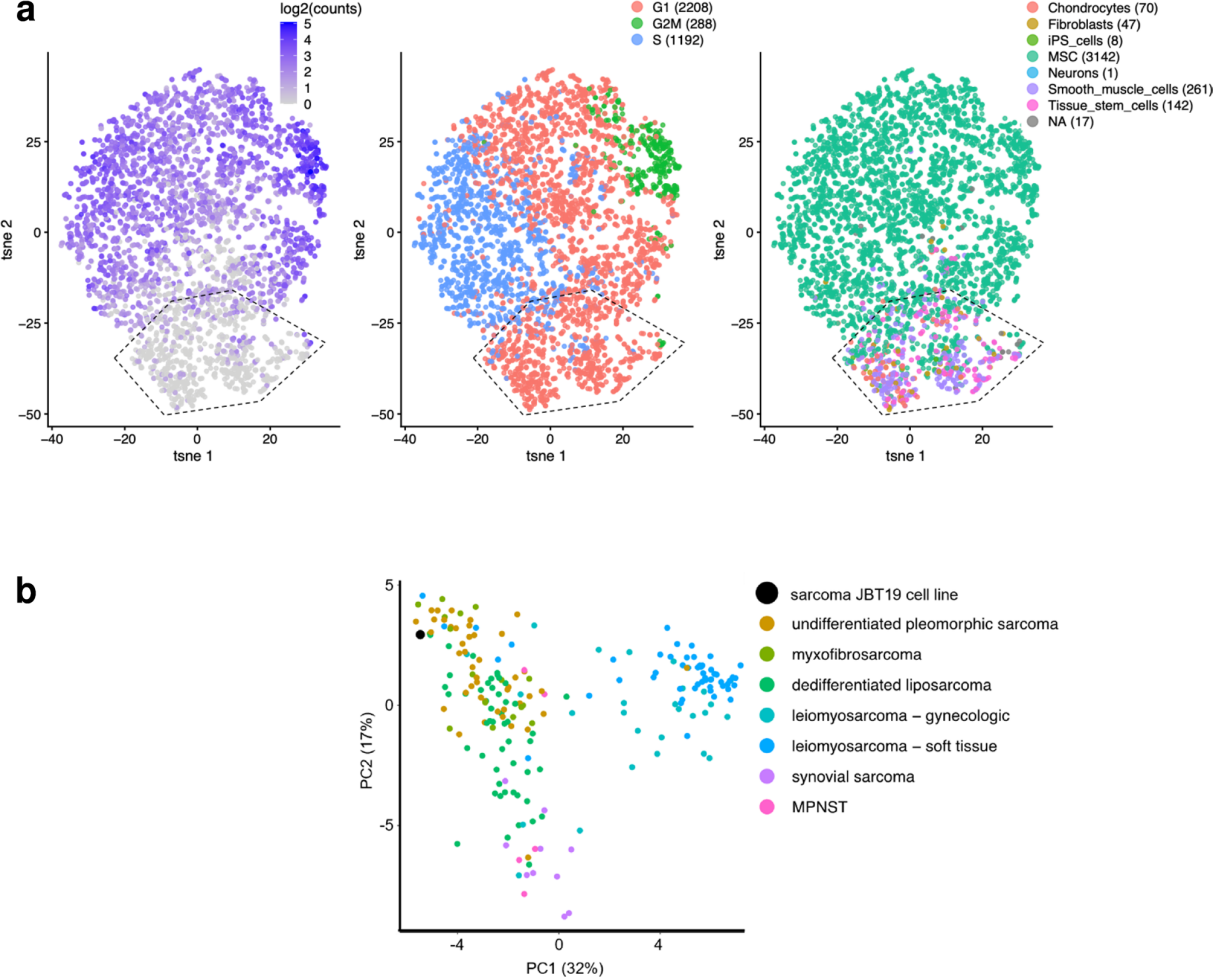
Transcriptome signatures of JBT19 cells. (**a**) The single-cell transcriptome profiling of 2D-cultured JBT19 cells shows the homogeneity of the JBT19 cell population. The cells do not show any significant stratification, with the exception of a weakly distinguishable subpopulation marked by the hexagon in the bottom left part of the t-distributed stochastic neighborhood embedding (tSNE) plot. These cells express less the marker of proliferation MKI67 (left), tend to be in the G1 phase of the cell cycle (center), and have blurry cell annotation (right, *iPS* induced pluripotent cells, *MSC* mesenchymal stem cells, *NA* not annotated). (**b**) When combined in a pseudo bulk sample, the JBT19 cells display the expression profile that matches the undifferentiated pleomorphic sarcoma or myxofibrosarcoma samples from the TCGA SARC study. These sarcoma subtypes are hardly distinguishable by molecular markers^35^. Each point represents one clinical sarcoma sample; MPNST stands for malignant peripheral nerve sheath tumor.

Many tumors are genetically highly heterogeneous and can contain multiple cell populations with diverse gene expression profiles^32,33^. Therefore, we investigated the diversity of gene expression in individual cells using single-cell sequencing of 2D-cultured JBT19 cell line in vitro. We found that the cells are in the G1, G2M, and S phases of the cell cycle and are most often annotated as *Mesenchyme stem cells (MSC)* in the *Human primary cell atlas*^34^. The cells do not show any significant separation in distinct populations. The only exception is a weakly distinguishable subpopulation of cells with low transcription activity of the marker of proliferation, the MKI67 gene. The majority of these cells are in the G1 phase of the cell cycle, and their cell-type annotation is fuzzy, ranging from *Tissue stem cells* to *Fibroblasts*, *Smooth muscle cells* and *Chondrocytes* (Fig. 4a) (Data available in the ArrayExpress database under accession E-MTAB-12758, https://www.ebi.ac.uk/biostudies/arrayexpress/studies/E-MTAB-12758).

To further characterize the JBT19 cell line, we combined the cells into a pseudo bulk sample and compared its expression profile with the clinical samples from the TCGA SARC study, which identified five sarcoma subtypes, so-called iClusters^35^. The expression profile of the iCluster markers clearly classifies JBT19 in the iCluster 5 (Fig. 4b, Supplemental Fig. S2), which represents mainly the samples of undifferentiated pleomorphic sarcoma and hardly distinguishable myxofibrosarcoma.

### JBT19 spheroids and in vivo mouse xenograft model

**Figure 5:**
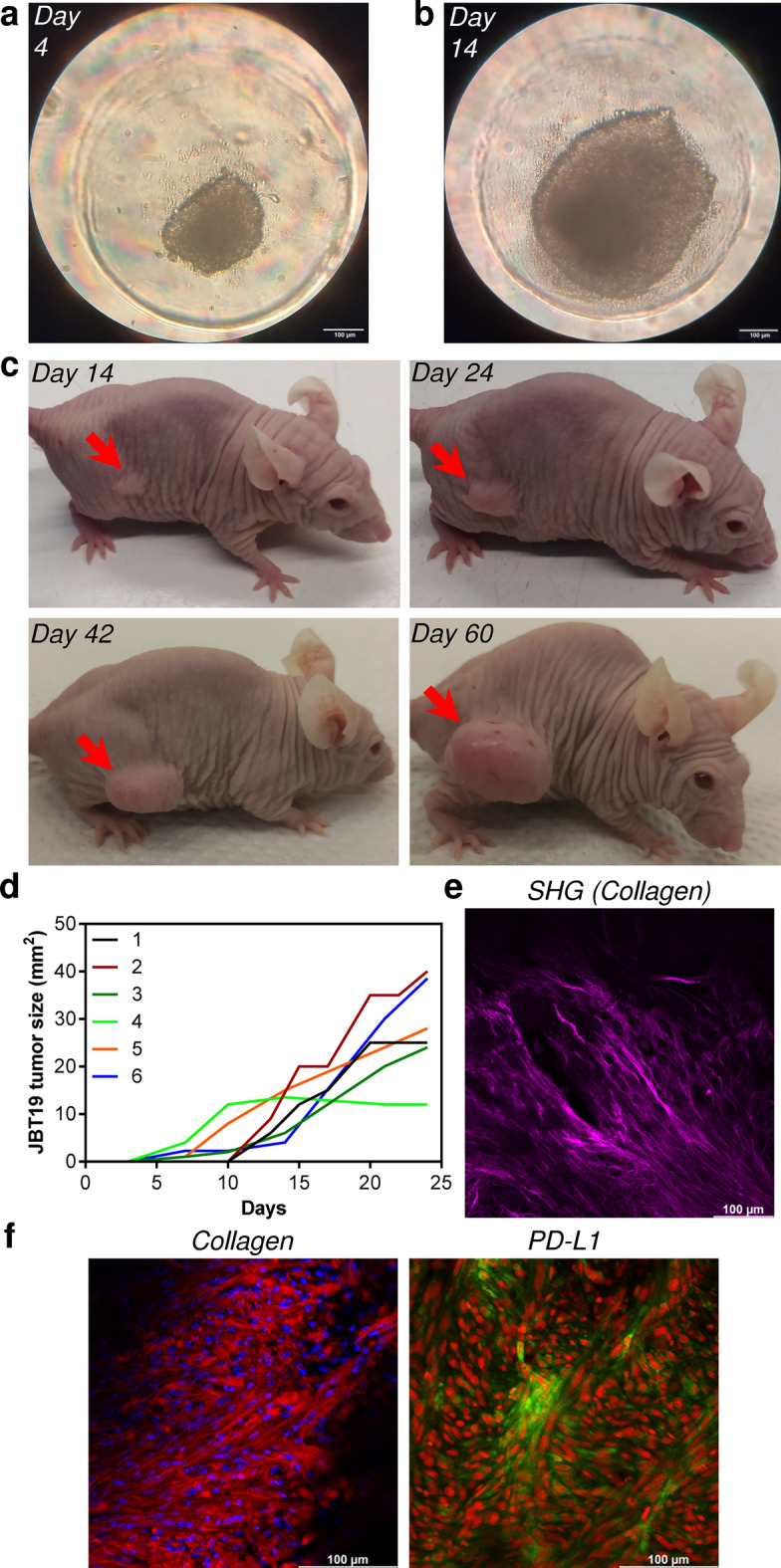
JBT19 spheroids and characteristics of JBT19 tumors developed in nude mice. (**a**,**b**) Representative images of a 4-day (**a**) and 14-day (**b**) spheroid originated by seeding of 0.273 × 10^6^ JBT19 cells/agarose 81-microwell dish at day 0. (**c**) Nude mice after s.c. inoculation of JBT19 cells (1 × 10^6^). Representative images of the development of a localized JBT19 tumor (*red arrow*) in a nude mouse 14 (top left panel), 24 (top right panel), 42 (bottom left panel), and 60 (bottom right panel) days after the inoculation. (**d**) Growth of JBT19 tumors after s.c. inoculation of JBT19 cells (1 × 10^6^) in nude mice (*n* = 6). (**e**) Collagen distribution and organization in 24-day-old JBT19 tumor sections imaged by two-photon confocal microscopy in SHG mode. (**f**) Representative visualization of collagen structures (left panel) and PD-L1 (right panel) in 24-day JBT19 tumor sections using immunohistochemistry staining with specific antibodies and one-photon confocal microscopy. In (**f**), collagen is visualized in red, PD-L1 is visualized in green; nuclei are stained with Hoechst 33,258 (blue) or Draq5 (red). The bars in (**a**,**b**) and (**e**,**f**) are 100 μm.

The JBT19 cells in 2D culture were observed to tend to pile up when overgrown. We, therefore, tested whether the cells could form and grow into three-dimensional (3D) structures (spheroids). Using a 3D cell culture model, we found that seeding 0.273 × 10^6^ JBT19 cells in agarose micro-well dishes (see “Methods”) and culturing them for 4 days led to the formation of spheroids with 250–300 μm in diameter (Fig. 5a), and after 14 days they reached the size of 400–600 μm in diameter (Fig. 5b). These data showed that the 3D cultivation of JBT19 cells was able to generate stably growing tumor spheroids. This finding indicated that JBT19 cells could also have the potential to become xenografted in immunocompromised animal models to form solid tumors. To test this potential, 1 × 10^6^ JBT19 cells were subcutaneously inoculated in 6 athymic nude mice (nu/nu). Initially, 2 mice were followed for 60 days after the s.c. inoculation of the sarcoma cells, as a preliminary observation of the growth potential. As shown in Fig. 5c, the inoculated JBT19 cells demonstrated development of progressively growing solid tumor in the nude mouse. The capability of JBT19 cells to form solid tumors was then confirmed in other 4 nude mice followed for 24 days. The tumors were stably growing, as shown in Fig. 5d, comprehensive of all observed mice until the 24^th^ day of the experiment. These tumors were found to contain the collagen tissue structures as determined by two-photon confocal microscopy in the second harmonic generation mode (SHG)^36^ (Fig. 5e). The collagen and PD-L1 expression in the JBT19 tumors was then confirmed by staining with specific antibodies and visualization with confocal microscopy (Fig. 5f).

### Ex vivo immunogenicity of JBT19 cells

**Figure 6:**
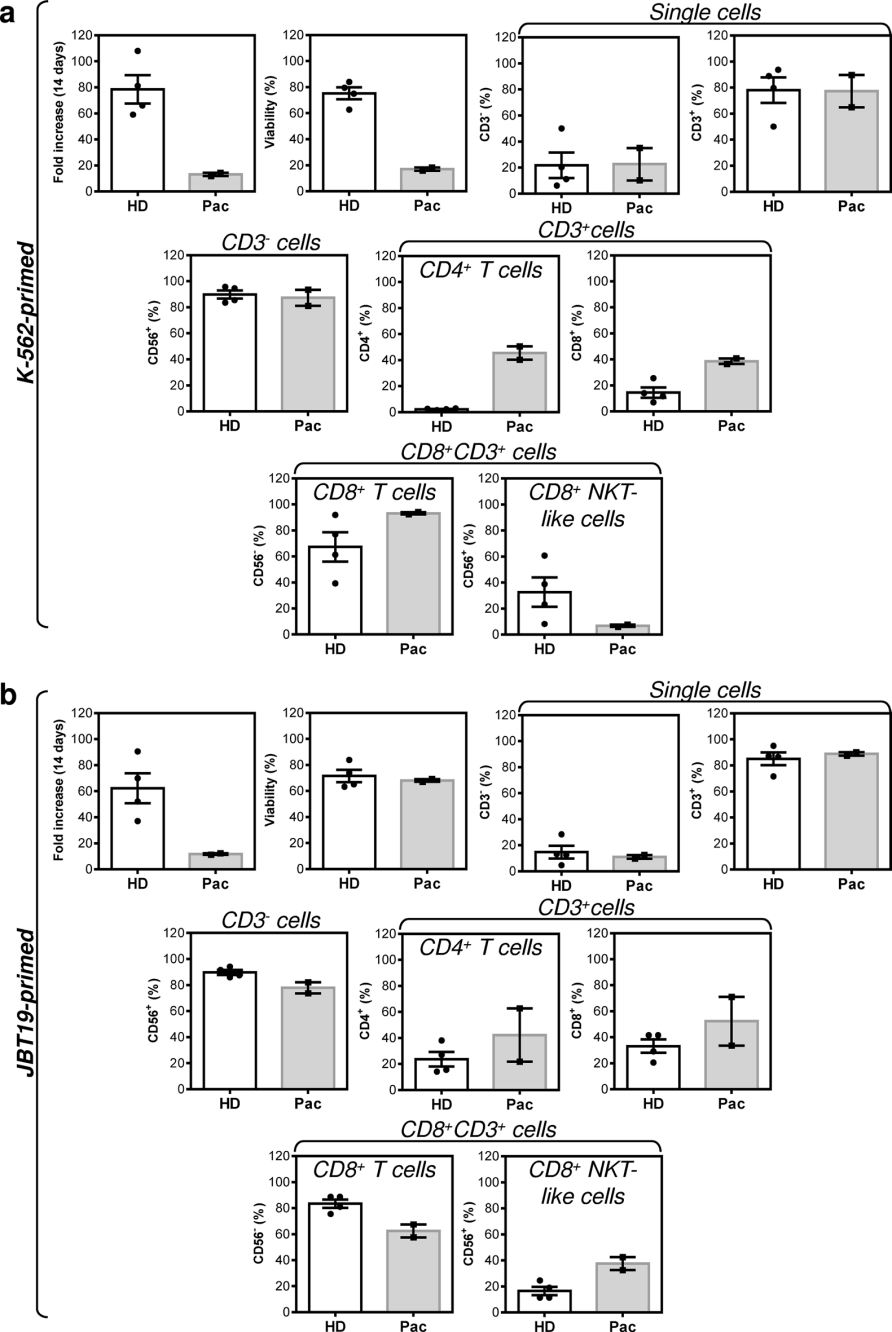
Characterization of K-562- or JBT19-primed and expanded healthy donors' and patient's lymphocytes (**a**) Proliferation, viability, and lymphocyte population frequencies of the K-562-primed and expanded healthy donors' (HD) and patient's (Pac) lymphocytes determined using the gating strategy in Fig. 7a. (**b**) Same tests like in (**a**) but using JBT19-primed and expanded HD and Pac lymphocytes. Bars represent the mean of values and SEM determined in each group (HD, *n* = 4 donors; Pac, *n* = 2 preparations).

**Figure 7:**
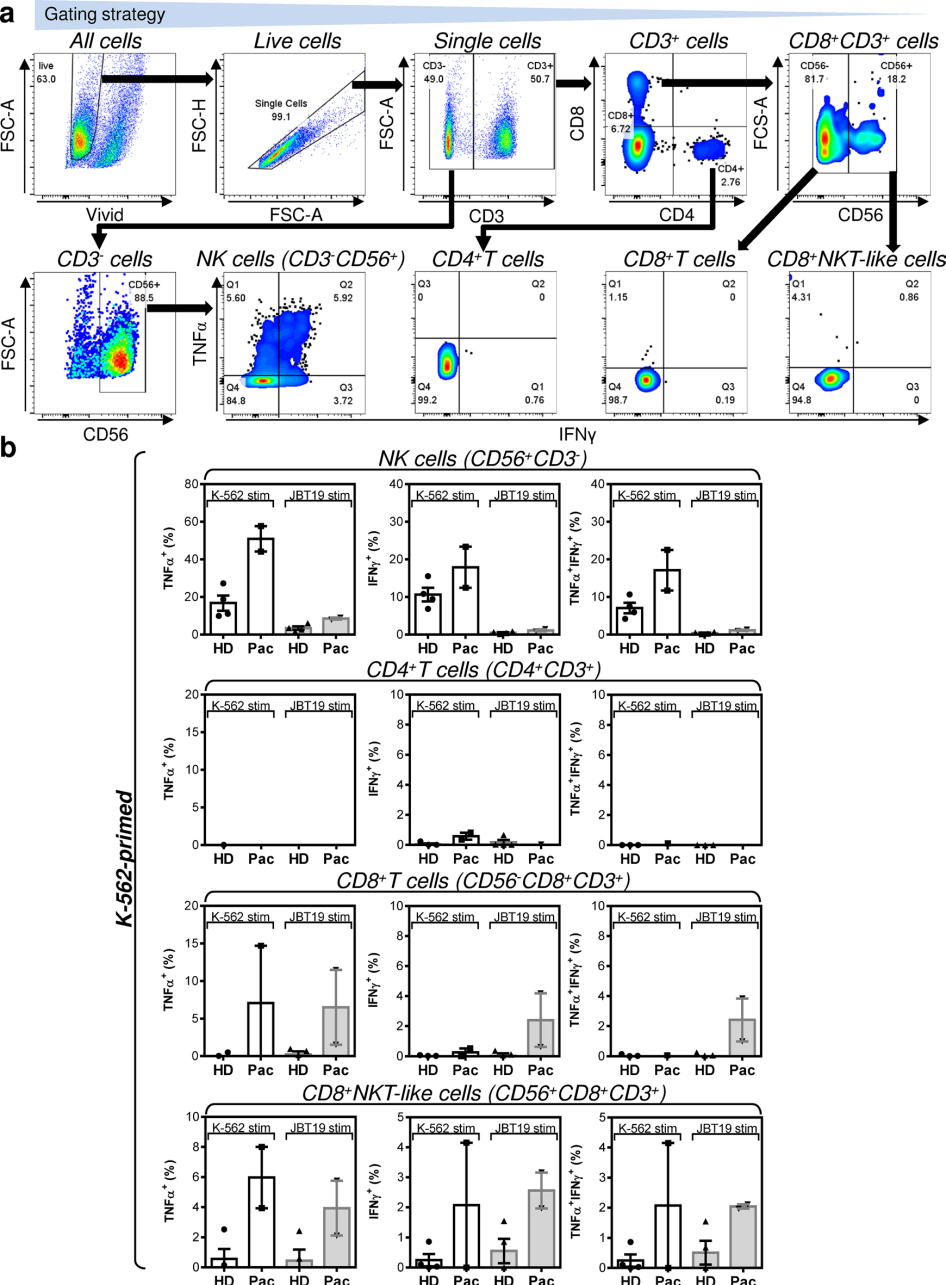
Reactivity of K-562-primed and expanded healthy donors' and the patient's lymphocytes to K-562 and JBT19 cells. (**a**) The gating strategy of flow cytometry data. (**b**) The frequencies of K-562- or JBT19-reactive TNFα^+^- (*left panels*), IFNγ^+^- (*middle panels*), and TNFα^+^/IFNγ^+^- (*right panels*) producing K-562-primed and expanded healthy donors' (HD) or patient's (Pac) lymphocyte populations after stimulation with K-562 (K-562 stim) or JBT19 (JBT19 stim) cells. Bars represent the mean of values and SEM determined in each group (HD, *n* = 4 donors; Pac, *n* = 2 preparations).

Immunotherapy of STSs has not so far shown encouraging results^37^. However, anti-PD-1/PD-L1 immunotherapy has shown promising results for specific histological subtypes^38^, among which is also UPS^39^. Together with high diversity in the tumor immune microenvironment^40,41^ and an overall high mutational burden of undifferentiated sarcomas^26^, UPS could be a promising model for investigating novel immunotherapeutic approaches. Therefore, we attempted to learn whether newly established JBT19 cells could be used for immunotherapy research on UPS. For this purpose, we used our previously developed protocols^42^ and customized them for ex vivo production of JBT19-reactive lymphocytes (details in “Materials and methods” section). We first investigated whether JBT19 cells could be in vitro challenged by immune cells. We used allogeneic PBMCs of healthy donors or autologous PBMCs of the patient as the source material of the immune cells. To prepare cancer cell-challenging immune cells, we expanded PBMCs in cell culture using the K-562 cell line in combination with IL-2. K-562 cell line is blasts established from a patient with chronic myelogenous leukemia (CML)^43^. These cells lack HLA-antigen expression and are used for ex vivo expansion and stimulation of NK cells^44,45^. Using inactivated K-562 cells and IL-2, we stimulated and expanded cultured PBMCs ex vivo (Fig. 6a, top left panel). Flow cytometry analysis (the gating strategy in Fig. 7a) showed that the expanded cell cultures contained NK cells (CD56^+^CD3^-^ cells), CD4^+^ and CD8^+^ T cells (CD3^+^), and CD8^+^ NKT-like cells (CD56^+^CD8^+^CD3^+^ cells^46^) (Fig. 6a, Fig. S3a). We found that the expanded cells contained populations of NK, CD8^+^ T, and CD8^+^ NKT-like cells that were reactive to either K-562 or JBT19 cells. The reactivity was determined by intracellular production of TNFα and IFNγ in the stimulated cells (Fig. 7b). No notable K-562 or JBT19 reactivity was found in the expanded CD4^+^ T cells (Fig. 7b).

**Figure 8:**
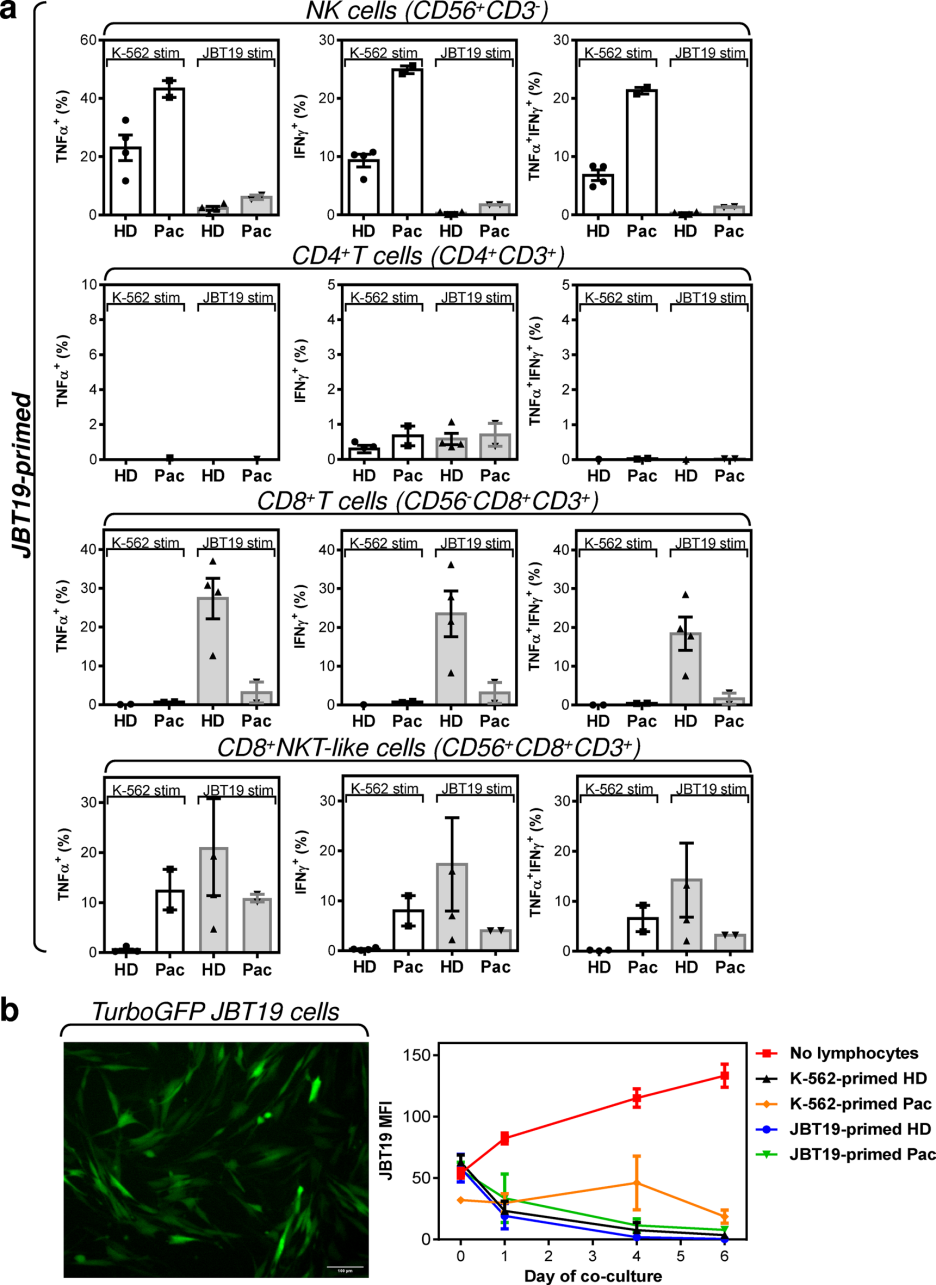
Reactivity of JBT19-primed and expanded healthy donors' and the patient's lymphocytes to K-562 and JBT19 cells. Cytotoxic impact of K-562- or JBT19-primed and expanded HD and patient's lymphocytes on 2D-cultured TurboGFP-JBT19 cells. (**a**) The flow cytometry data were gated as in Fig. 7a. Shown are frequencies of K-562- or JBT19-reactive TNFα^+^- (*left panels*), IFNγ^+^- (*middle panels*), and TNFα^+^/IFNγ^+^- (*right panels*) producing JBT19-primed and expanded healthy donors' (HD) or patient's (Pac) lymphocyte populations after stimulation with K-562 (K-562 stim) or JBT19 (JBT19 stim) cells. Bars represent the mean of values and SEM determined in each group (HD, *n* = 4 donors; Pac, *n* = 2 preparations). (**b**) Adherent TurboGFP-JBT19 cells (*left panel*) were cocultured with expanded HD and patient's lymphocytes, and the cytotoxic impact of the coculture on TurboGFP-JBT19 cells was evaluated via the TurboGFP fluorescence in the cell culture (*right panel*). Bars represent the mean of values and SEM determined in each group (HD, *n* = 4 donors; Pac, *n* = 2 preparations).

In the next step, we examined whether replacing K-562 cells with JBT19 cells for lymphocyte priming could also lead to the expansion of the JBT19- and/or K-562-reactive lymphocytes. We found that using JBT19 cells for the lymphocyte priming also led to cell culture expansion (Fig. 6b, top left panel). We confirmed that the expanded cell cultures contained NK cells, CD4^+^ and CD8^+^ T cells, and CD8^+^ NKT-like cells (Fig. 6b, Fig. S3b). Comparably to the expanded K-562-primed lymphocytes, the cell cultures contained populations of NK, CD8^+^ T, and CD8^+^ NKT-like cells that were reactive to either K-562 or JBT19 cells (Fig. 8a). No or very little reactivity was observed for CD4^+^ T cells (Fig. 8a).

In the next analyses, we investigated whether the observed JBT19-reactivity of the expanded lymphocytes was associated with any cytotoxic impact on JBT19 cells. To answer this question, we prepared JBT19 cells stably expressing TurboGFP fluorescent protein (Fig. 8b, left panel). Then we cocultured these cells with the K-562- or JBT19-primed and expanded lymphocytes and determined the content of the TurboGFP-JBT19 cells in the cell coculture as previously described^47^. We found that without lymphocytes, the TurboGFP-JBT19 cells expanded in the cell culture (Fig. 8b, right panel). However, when TurboGFP-JBT19 cells were cocultured with the expanded lymphocytes, their growth was arrested, or they were eliminated from the cell coculture (Fig. 8b, right panel). These data revealed that the expanded lymphocytes could deliver cytotoxic impact to the 2D-cultured JBT19 cells. These data show that the novel UPS cell line is immunogenic to both allogeneic and autologous lymphocytes. This immunogenicity thus opens opportunities for the JBT19 cells to be used in the research investigating immunotherapeutic interventions in the treatment of UPS.

### Ex vivo-produced JBT19-reactive cytotoxic lymphocytes are largely LAIR-1^+^

**Figure 9:**
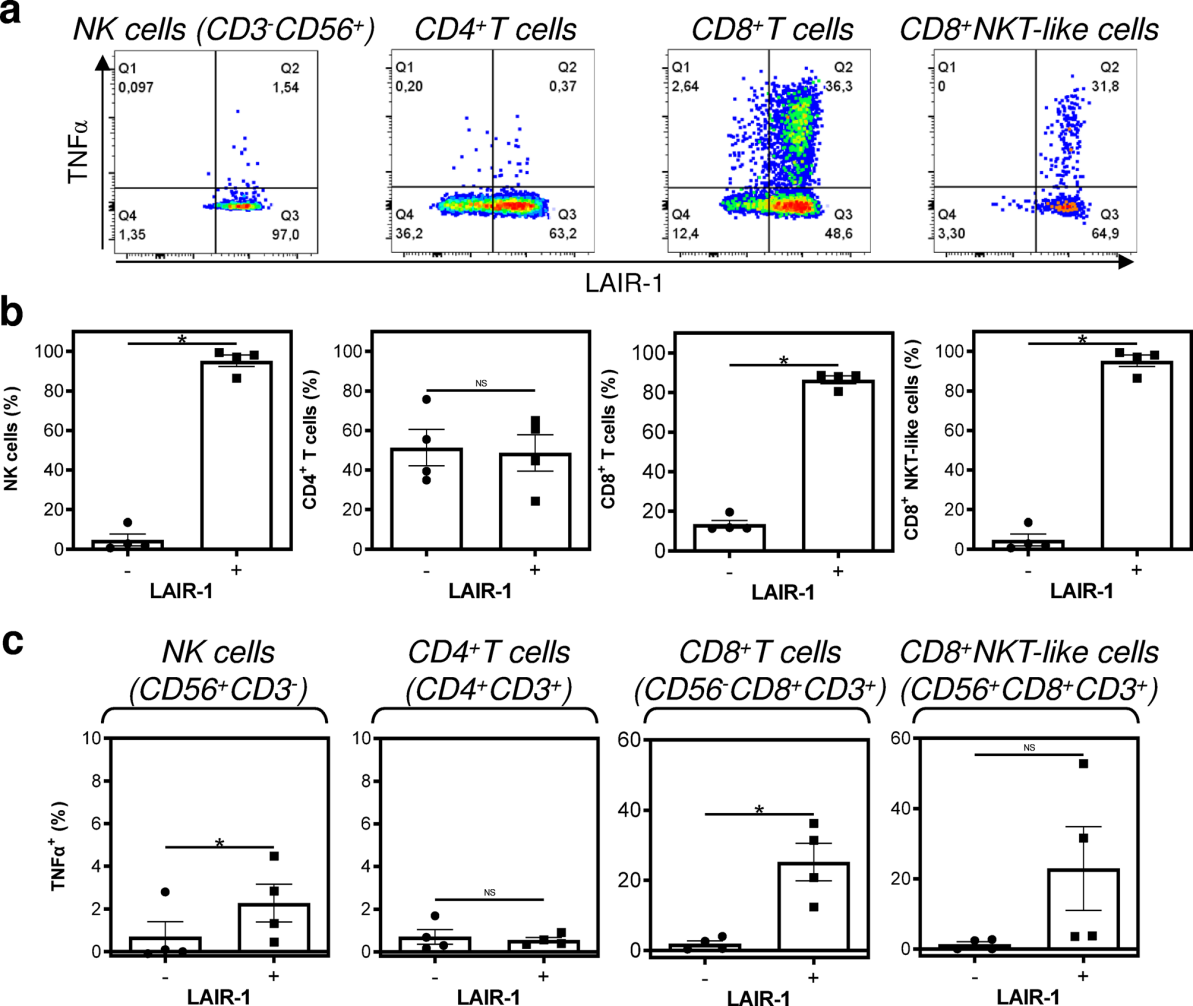
LAIR-1 expression in JBT19-primed and expanded healthy donors' lymphocytes after stimulation with JBT19 cells. (**a**) The flow cytometry data were gated as in Fig. 7a. Shown are representative images of JBT19-stimulated cells. (**b**) The frequencies of LAIR-1^-^ (*left bars*) and LAIR-1^+^ (*right bars*) JBT19-primed and expanded healthy donors' (HD) NK cells, CD4^+^ T cells, CD8^+^ T cells, and CD8^+^ NKT-like cells. (**c**) The frequencies of JBT19-reactive and TNFα-producing LAIR-1^-^ (*left bars*) or LAIR-1^+^ (*right bars*) JBT19-primed and expanded healthy donors' (HD) NK cells, CD4^+^ T cells, CD8^+^ T cells, and CD8^+^ NKT-like cells after stimulation with JBT19 cells. Bars in (**a**) and (**b**) represent the mean of values and SEM determined in each group, and statistical significances of differences between the group of LAIR-1^−^ and LAIR-1^+^ populations are indicated (*p < 0.05; HD, *n* = 4; paired two-tailed Student's t-test).

Collagen is a ligand of the immune checkpoint molecule LAIR-1^48^. We next investigated LAIR-1 expression in the ex vivo-expanded JBT19-primed lymphocytes of healthy donors (Fig. 9a). We found that CD4^+^ T cells of the expanded lymphocytes had comparable frequencies of LAIR-1^-^ and LAIR-1^+^ populations (Fig. 9b, second left panel). However, significantly higher frequencies of LAIR-1^+^ cells were found in NK cells, CD8^+^ T cells, and CD8^+^ NKT-like cells (Fig. 9b, left and two right panels). Stimulation of the expanded lymphocytes with JBT19 cells also revealed that, except for the CD4^+^ T cells and CD8^+^ NKT-like cells, the JBT19-reactive population (TNFα-producing) of NK cells and CD8^+^ T cells had significantly higher frequencies of LAIR-1^+^ populations than their LAIR-1^-^ counterparts (Fig. 9c). These data showed that JBT19 cells could not only form solid tumors in the animal model but also be used for ex vivo production of JBT19-reactive lymphocytes with predominant expression of the inhibitory molecule LAIR-1.

## Discussion

In this study, we describe a novel and, at present, unique patient-derived UPS cell line JBT19, which expresses PD-L1 and collagen, respectively, ligands of the currently intensively studied two checkpoint molecules, PD-1 and LAIR-1. The cell line was well growing in vitro in both 2D- and 3D-cell cultures and produced solid tumors when a cell suspension was subcutaneously inoculated in nude mice. The cell line was also immunogenic to both allogeneic and autologous lymphocytes and could be used for ex vivo production of LAIR-1^+^ JBT19-reactive lymphocytes.

Cell lines are widely used for in vitro and in vivo models to investigate the biological behavior of tumor cells and to investigate the mechanisms of their resistance to therapy. Many of UPS cell lines were established in multiple studies in previous years^49–52^. However, although the establishment of new UPS cell lines is ongoing^52^, there are still not only a limited number of UPS cell lines available through public depositories^17^, but, to our knowledge, none of the studies previously published directly addressed and investigated their suitability for immunotherapeutic research. The newly described JBT19 cell line in this study was not only largely described on the molecular level and found suitable for both in vitro and in vivo studies but also shown to be suitable for immunotherapeutic research.

Transcriptome profiling and validation of UPS markers showed that the expression profile of the JBT19 cell line has features of UPS cells from clinical samples^35^. Cytogenomic analyses also confirmed multiple defects in the karyotype. These defects are typical for UPS^26^, and their findings in JBT19 cells confirmed that the cells were indeed transformed cells. In addition, UPS is known for its aggressive behavior. Although this behavior can have different degrees due to the high heterogeneity of UPS and the microenvironment in which they develop, the cytogenomic analyses revealed the deletion of the *CDKN2A* gene in JBT19 cells. The *CDKN2A* gene deletion can define aggressive behavior of transformed cells^53^ and is often associated with poor prognosis of many cancers^54,55^, including STSs^30^. The deletion of the *CDKN2A* gene in JBT19 cells may therefore suggest their aggressive behavior, the extent of which could also be corroborated by the CD44 molecule, which is also expressed in JBT19 cells and which is also associated with worse STS outcomes^56^. However, how much the behavior of JBT19 cells is indeed aggressive needs more extensive investigation in animal models alongside therapeutic interventions.

The suitability of the JBT19 cell line for immunotherapeutic research lies in the expression of molecules largely considered or already widely used as immunotherapeutic targets. The suitability also lies in the demonstration that JBT19 cells are immunogenic to both allogeneic and autologous lymphocytes. Therefore, using the presented cell culture protocol, JBT19-reactive lymphocytes could be ex vivo produced for therapeutic interventions to be tested in experimental models, even in association with other (immune) therapeutics, in the view of clinical applications.

One of the PD-1 ligands expressed on many tumor cells and many established cell lines is PD-L1. PD-L1 belongs to a few of the most targeted molecules in cancer immunotherapy^12^. However, targeting this molecule is not always successful in solid tumors^57^. This failure is often due to multiple mechanisms^57^, including the co-expression of other inhibitory molecules^58^. One of the molecules solid tumors broadly use against immune cells is collagen^59^. Collagen forms not only a physical barrier that can protect the tumor from immune cell infiltration, but it is also a ligand for inhibitory receptors expressed on the surface of immune cells. One of these molecules is LAIR-1. LAIR-1 is a recently described novel immune checkpoint molecule expressed on immune cells^60^. It interacts with collagen, and cells expressing LAIR-1 bind strongly to collagen^48^. LAIR-1-mediated signaling suppresses T cell activation^61^ and elicits immunosuppression^18,21^. Our analyses showed that 2D- and 3D-cultured JBT19 cells express collagen, and once these cells are engrafted in nude mice, they form high-collagen-content solid tumors. Since these tumors also express PD-L1, JBT19 cells represent a unique model for investigating PD-1 and LAIR-1 interplay in solid tumor resistance. This uniqueness is particularly relevant in light of recent findings showing that collagen promotes anti-PD-1/PD-L1 resistance in cancer through LAIR1-dependent CD8^+^ T cell exhaustion^21^.

The inhibitory impact of the collagen/LAIR-1 axis on the immune cells indicates that research on immunotherapy, namely ACI and ICI immunotherapy, could be the primary area of use for JBT19 cells. Results of this study showed that JBT19-reactive immune cell populations could be ex vivo generated from both allogeneic and autologous lymphocytes. Whereas the ex vivo generation of the JBT19-reactive allogeneic immune cells was presumably corroborated by HLA-unmatched recognition on JBT19 cells, the ex vivo generation of autologous JBT19-reactive immune cell populations was certainly the result of the ability of the patient's immune cells to recognize the transformed cells, presumably via the tumor-associated or -specific antigens^62,63^. And indeed, we were able to ex vivo demonstrate the generation of JBT19-reactive NK cells, CD8^+^ T cells, and CD8^+^NKT-like cells. Specifically, CD8^+^NKT-like cells were recently found to exert dual toxicity, killing in an antigen-specific manner not only tumor cells but also myeloid-derived suppressor cells^64^. Our data indeed confirmed that ex vivo-generated lymphocytes containing JBT19-reactive immune cell populations were able to exterminate JBT19 cells in 2D cell coculture. In addition, although it remains to be determined which of the JBT19-reactive effector cells could deliver the cytotoxic impact, our data also revealed that these JBT19-reactive immune cells were largely expressing LAIR-1 which suggests that the efficacy of their anti-JBT19 cytotoxic activities could be challenged in vivo in the high-content collagen JBT19 tumors, either via collagen itself or in association with other LAIR-1 agonists expressed in the tumor microenvironment. Therefore, these findings showed that the utility of JBT19 cells in immunotherapeutic research could not only lie in the formation of high-content collagen solid tumors in animal models where (immune)therapeutic interventions could target these cells but also in their ability to ex vivo produce LAIR-1^+^ JBT19-targeting effector immune cells suitable for the treatment of JBT19 tumors to directly challenge and investigate the collagen/LAIR-1 inhibitory mechanisms^18,20,21,48^.

The traditional treatment efficacy of STSs is generally very low^46^. Immunotherapy has been attempting to step in recent years, namely relying on anti-PD-1/PD-L1 blocking antibodies^16^. Further development of immunotherapeutic approaches is, however, limited due to a limited number of UPS cell lines^17^. Moreover, recent studies showing the importance of LAIR-1 in the regulation of tumor resistance to anti-PD-1/PD-L1 immunotherapy stress the importance of studying the collagen/LAIR-1 inhibitory axis^21^, particularly in the context of high-content collagen tumors^59^. In the attempt to break the solid tumor resistance, novel therapeutic protocols largely rely on combined therapies, including combinations of different immunotherapeutic approaches^65,66^. These combinations could be well investigated using JBT19-based in vivo models. These combinations may include PD-1/PD-L1 and LAIR-1 checkpoint inhibitors^67^, or even their combinations with adoptive transfer of ex vivo-produced JBT-19-reactive LAIR-1^+^ lymphocytes or PD-1-expressing lymphocytes that could be produced ex vivo using, for instance, monocyte-derived dendritic cells in combination with LL-37^68^. In addition, our phenotypic analysis of JBT19 cells revealed the expression of other currently promising immunotherapeutic targets, some of which are already tested in or considered for clinical trials—CD47^69,70^, FAP^71^, and CD95^72^. From this perspective, the PD-1/PD-L1 and collagen/LAIR-1 inhibitory axes in JBT19 tumors could be interrogated alongside other mechanisms of solid tumor resistance and therapeutic interventions.

Our results consistently showed that JBT19 human sarcoma cells stably express PD-L1, can in vivo produce high-content collagen tumors, and ex vivo be used to produce LAIR-1^+^ JBT19-reactive lymphocytes. Thus combined, the JBT19 human sarcoma cells can offer a novel and unique study model for immunotherapy research on UPS.

## Materials and methods

### Patient history

The patient donor was a 65-year-old caucasian male at the time of diagnosis in August 2019. The patient presented in the Motol University Hospital with an unknown soft tissue mass that developed in the thigh muscle. The tissue mass (18 × 14 × 5 cm) was resected during the surgery. The tumor mass was then split for pathological examination and further processing at the Department of Immunology. The pathological examination confirmed the presence of a solid tumor mass (8 × 5 × 8 cm) diagnosed as Grade 3 UPS. The patient provided a prior signed written informed consent for using a portion of the excised tissue for future research. The research was approved according to the ethical standards of the institutional research committee—the Ethics Committee of the Motol University Hospital in Prague (approval date: Jun 19, 2019), and performed in compliance with the 1964 Helsinki declaration and its later amendments or comparable ethical standards.

### Cell line establishment

The portion of the whole excised tissue mass was rinsed several times with a cell culture medium [LM medium; RPMI 1640 medium (Thermo Scientific, Waltham, MA, USA) containing 5% human serum (One Lambda, Canoga Park, CA, USA), 100 U/mL penicillin–streptomycin, 2 mM GlutaMax, 1 mM sodium pyruvate, and nonessential amino acid mix (Thermo Scientific)]. A portion of the rinsed tissue mass was used for the cell line establishment. The JBT19 cell line was established in cell cultures using a combination of tumor fragmentation and dissociation techniques previously described^22–25^. The fragmented pieces were transferred into a 6-well flat-bottom plate (TPP, Trasadingen, Switzerland) and supplemented with LM medium. The plate with the processed material was then cultured (37 °C, 5% CO_2_). The medium was half-depleted every second or third day and supplemented with fresh LM medium. After 8 days of cell culture, the non-adherent fraction was removed from the plate, and the adherent cells were rinsed with PBS and harvested after the treatment with trypsin/EDTA solution (Thermo Scientific). The cell suspensions were pelleted by centrifugation and passaged into T75 tissue flasks in LM medium (TPP). The cells were cultured as adherent monolayers (2D cell cultures) for over 30 months and found to be well cryopreserved in LM medium with 10% DMSO and well reconstituted back in the cell culture after storage in liquid nitrogen. The cryopreserved aliquots prepared after 30 passages and after over 30 months in the cell culture were then deposited in the European Collection of Authenticated Cell Cultures (Accession number given by the International Depository Authority: 22,113,001, date: Dec 15, 2022).

### Microscopy

For fluorescence immunohistochemistry, we used 2D-cultured JBT19 cell lines or JBT19 tumors developed in nude mice. With some modifications, the samples were processed as described previously^73^. Briefly, the adherent JBT19 cells were collected to be seeded in an 8-well plate with a thin bottom glass (C8-1.5H-N, Cellvic, Mountain View, USA) for confocal microscopy observation. The expansion of the cells was checked with an inverted microscope (Meopta, Prerov, Czech Republic). After expansion, cells were washed with PBS and fixed in a 4% paraformaldehyde solution (Thermo Scientific). After fixation, the cells were washed with PBS and permeabilized with Triton X-100 (MP Biomedicals, Illkirch, France). Then cells were washed with PBS and incubated for 1 h in 2% bovine serum albumin (Sigma-Aldrich, St. Louis, MO, USA) at room temperature. Subsequently, the wells were rinsed with PBS and stained with fluorescently labeled antibodies: PD-L1 (CD274)-APC (clone MIH1, Human) (eBiosciences, San Diego, CA, USA), PD-L1 (CD274)-FITC (clone MIH3) (Biolegend, San Diego, CA, USA), COL1A1 XP-AlexaFluor 647 (clone E8F4L) (Cell Signaling, Danvers, MA, USA). Either Hoechst 33,258 (Abcam, Cambridge, United Kingdom) or Draq5 (Biostatus, Shepshed, United Kingdom) were used to stain nuclei. The same was repeated for thin slices of fresh sarcoma tissue from nude mice. Samples were analyzed using one-photon Olympus FV1000 TIRF (Shinjuku City, Tokyo, Japan), and two-photon Bruker Ultima IntraVital (Billerica, Massachusetts, USA) confocal microscopy. The single-photon confocal microscopy used 405 nm (blue), 473 nm (green), and 635 nm (red) laser, and the acquired images were processed using the Olympus FLUOVIEW Ver.4.2 viewer software. For two-photon confocal microscopy, the Bruker Ultima IntraVital device in second harmonic generation mode (SHG) was used to visualize the collagen tissue structure in unstained fresh tissue sections using the 810 nm wavelength as described^36^. The acquired images were analyzed using the ImageJ software (NIH, MA, USA) and LAS X Leica (Wetzlar, Germany).

### Phenotypic characterization

For immunohistochemistry, the cells were processed as described^74^. The following primary antibodies were used: FAP (rabbit polyclonal NB100-59,021) (Novus Biologicals (Bio-Techne), Minneapolis, MN, USA), nestin (clone 10C2), and desmin (clone DE-U-10) (Abcam), and vimentin (clone V9) (Dako (Agilent), Santa Clara, CA, USA). As a secondary antibody was used Histofine^®^ Simple Stain™ MAX PO (MULTI) (Nichirei Bioscience Inc., Tokyo, Japan), and the chromogen was Histofine^®^ Simple Stain™ AEC Solution (Nichirei Bioscience Inc.). The bright field images were acquired using the Leica DM2000 microscope equipped with LASx software.

For the cell surface or intracellular expression profiling by flow cytometry, the adherent JBT19 cells were harvested by trypsinization and rinsed with LM medium and PBS with 2 mM EDTA (PBSE). The cells were stained as described^42^ with the following fluorescently labeled antibodies: CD95(Fas)-APC (clone DX2), CD178(FasL)-PE (clone NOK-1), CD31-PC7 (clone WM59), CD47-APC (clone CC2C6), CD263(TRAIL)-APC (clone RIK-2), CD279(PD-1)-APC (clone EH12.2H7), CD30-APC (clone BY88), Galectin-9-PE (clone 9M1-3), CD274(PD-L1)-APC (clone MIH3), HLA-ABC(MHC-I)-PE (clone W6/32), HLA-DP, DQ, DR(MHC-II)-APC (clone Tü39) (BioLegend), FAP-PE (clone 427,819), DR3-PE (clone 59,204) (R&D Systems, Minneapolis, MN, USA), CD117(c-Kit)-PE (clone YB5.B8) (Becton Dickinson, Franklin Lakes, NJ, USA), CD34-FITC (clone AC136), CD133/1-APC (clone AC133) (Miltenyi Biotec, Gladbach, Germany), CD44-PE (clone MEM-263) (Exbio, Prague, Czech Republic). For the intracellular staining was used: CD274(PD-L1)-APC (clone MIH3), αSMA-APC (clone 1A4) (BioLegend), COL1A1 XP-AlexaFluor647 (clone E8F4L) (Cell Signaling), vimentin-FITC (clone V9), nestin-AF488 (clone 10C2) (eBiosciences), and Ki-67 (clone Ki-67) (Exbio). As negative controls were used CD366(Tim-3)-PE (clone F38-2E2) and CD178(FasL)-PE (clone NOK-1) (BioLegend). The stained cells were analyzed using flow cytometry (FACSAria II or FACSFortesa, Becton Dickinson, Heidelberg, Germany), and the FlowJo software (Tree Star, Ashland, OR) was used to analyze the data.

### Cell line validation and HLA-genotyping analyses

Cell line validation was performed by genetic testing. Genomic DNA from cells was isolated using MagCore^®^ Genomic DNA Whole Blood Kit (RBC Bioscience, Taipeh, Taiwan) on the principle of paramagnetic beads in the presence of chaotropic salts according to the manufacturer's instructions. The validation of JBT19 cells was determined using genotyping of Short Tandem Repeats (STRs) polymorphisms by fragment analysis using the PowerPlex^®^ 16 HS System kit (Promega, Madison, WI, USA). HLA-genotyping was performed by Next Generation Sequencing using AlloSeq Tx17kit (CareDx, Brisbane, CA, USA). The obtained sequences were analyzed by AlloSeqAssignv.1.0.3.1331, IMGT/HLA database3.45.1.1 (accessed 2021-07-12).

### Karyotype analysis

Prior to harvest, the adherent JBT19 cells in the cell culture were treated with demecolcine solution (Sigma-Aldrich) for 16 h. Suspension of cells was obtained using short-termed trypsin treatment. Harvesting and preparation of slides were performed according to standard cytogenetic procedures. Harvested cells were stored at − 20 °C in methanol-glacial acetic acid (3:1 ratio). For the cytogenomic analyses, cell suspensions were dropped on microscopic slides and air-dried. Multicolor fluorescence in situ hybridization (mFISH) and interphase fluorescence in situ hybridization (I-FISH) were used to characterize the chromosomal aberrations in detail. The mFISH analyses were performed using a commercially available probe 24XCyte Multicolor FISH Probe (MetaSystems, Altlußheim, Germany). All available metaphases were scanned using Metafer AxioImager Z2—automatic metaphase finder and AxioImager Z1 fluorescence microscope (Carl Zeiss, Jena, Germany) and further analyzed using Isis computer analysis system (MetaSystems). I-FISH analyses were performed using commercially available locus-specific probes: the XL CDKN2A Dual Color probe (MetaSystems, Altlußheim, Germany) to detect deletions in band 9p21 and the Vysis LSI 13 (RB1) 13q14 Spectrum Orange/LSI 13q34 SpectrumGreen probe (Abbott Vysis, Illinois, USA) to detect deletions in band 13q14. Two hundred interphase nuclei for both probes were analyzed using an AxioImager Z1 fluorescence microscope (Carl Zeiss, Jena, Germany) and the Isis computer analysis system (MetaSystems, Germany). The findings were described according to the international system for human cytogenomic nomenclature (ISCN) 2020^27^.

### Transcriptome profiling

The 2D-cultured JBT19 cells for transcriptomic profiling at the single cell level were prepared as described elsewhere^74^. Briefly, cells were seeded at 10,000 cells/cm^2^ and cultured for 72 h in T25 culture flasks. To prepare cell suspension, the cells were thoroughly washed with PBS and rapidly harvested in a 0.25% trypsin + EDTA solution mixture (at a 1:1 ratio) (Sigma-Aldrich). The viability of cells was assessed by trypan blue and counted in an automated TC20 cell counter (Bio-Rad, Hercules, CA, USA). The sample had cell viability above 80%.

Single-cell RNA-seq libraries were prepared using a Chromium controller instrument and Chromium Next Gem single-cell 3’ reagent kit (version 3.1) according to the manufacturer’s protocol (both 10 × Genomics, Pleasanton, CA, USA) targeting 4000 cells per sample. The quality and quantity of the resulting cDNA and libraries were determined using an Agilent 2100 Bioanalyzer (Agilent Technologies, Santa Clara, CA, USA). The library was sequenced on a NovaSeq 6000 sequencer (Illumina, San Diego, CA, USA) by SEQme company (Dobris, Czech Republic) according to the manufacturer’s protocol yielding on average 45 thousand reads per cell. Sample demultiplexing of raw data from Illumina NovaSeq 6000 sequencer was performed by cellranger software (v4.0.0) provided by 10 × Genomics.

Raw sequencing data were processed by CellRanger software v. 4.0.0 (10 × Genomics, Pleasanton, CA, USA). The resulting raw feature barcode matrices were analyzed using the *scdrake* pipeline (https://github.com/bioinfocz/scdrake, Ref.^75^). Empty droplets containing only ambient RNA were removed using *DropletUtils*^76^. Subsequently, dead or damaged cells were filtered out, and features expressed in less than 5% of cells were removed from the barcode matrix, resulting in 3688 cells and 12,222 features. The cell cycle phase was assessed using the *SeuratCellCycleScoring*^77^ function, and tentative cell annotation was performed using *SingleR*^78^ with *celldex* annotation base *HumanPrimaryCellAtlasData* derived from Ref.^34^.

All transcriptomic data were deposited in the ArrayExpress database (https://www.ebi.ac.uk/biostudies/arrayexpress) under accession E-MTAB-12758.

### Validation of UPS markers

To validate that the JBT19 cell line expresses markers typical for UPS, we used the SARC dataset from The Cancer Genome Atlas as a reference. The original study identified five molecular subtypes of sarcoma, so-called iClusters. UPS samples were classified mainly in iCluster 5^35^.

For TCGA SARC clinical samples, raw bulk RNA-seq counts were downloaded from the XENA platform (Ref.^79^, GDC TCGA SARC dataset, only primary tumors were analyzed) and transformed into TPM counts. To obtain pseudo-bulk RNA-seq of the JBT19 cell line, we filtered raw UMI count matrix from our scRNA-seq experiment for empty droplets and summed counts over genes. The pseudo-bulk counts were then transformed to TPM. The TPM values were log-transformed, and gene-wise z-score was calculated. The visualization represented in the heatmap in Supplemental Fig. S2 is based on the overlap of genes detected in our scRNA-seq dataset and the top twenty genes with the highest mean expression in each iCluster in the original article^35^. The same data was used in the principal component analysis of Fig. 4b.

### 3D cell culture model

The cells were 2D-cultured in LM medium (37 °C, 5% CO2) for at least 3 days. After reaching 90% coverage of the culture area, the cells were washed with PBS and harvested using trypsin/EDTA. To produce spheroids, the Microtissues 3D Petri Dish™ technology (Sigma-Aldrich) was used. The system is based on a mold (cat. #12-81) used to create a 3D Petri Dish™ from agarose (1.5% PCR agarose, Top-Bio, Czech Republic). On the gel, 0.273 × 10^6^ JBT19 cells were seeded in 190 μl of LM medium and then cultured (37 °C, 5% CO2) for 14 days, with microscopy follow-up of their development.

### In vivo mouse xenograft model

For in vivo studies, 6 athymic nude mice (nu/nu) (AnLab, Prague, Czech Republic) were used. The mice were inoculated with JBT19 cells (1 × 10^6^ s.c.), and the tumor growth was followed over the established observation period by evaluation every 2–3 days of the tumor dimensions by a caliper. For the microscopic analyses, the mice (*n* = 6) were sacrificed by cervical dislocation, and the developed JBT19 tumors were resected, sliced, and processed for microscopy as described above. The animal studies were approved by Project No. 111/2019. The studies were approved by the Departmental Commission of the Academy of Sciences of the Czech Republic, according to Act of the Czech National Council no. 246/1992 Coll., and according to § 5 of Decree no. 419/2012 Coll., the Act on the Protection of Animals Against Cruelty under Project No. 111/2019. This study is reported according to the ARRIVE guidelines (https://arriveguidelines.org). The standard housing conditions and the experimental procedures respected the rules of the European Convention for the Care and Use of Laboratory Animals (2010/63/EU) as approved by the Czech Animal Care and Use Committee and by the Institutional Ethics Committee.

### Preparation of autologous and allogeneic JBT19-reactive lymphocytes

The source material for the preparation of autologous JBT19-reactive lymphocytes was unclotted peripheral blood collected during the planned health check-up of the donor UPS patient. The source material for the preparation of allogeneic JBT19-reactive lymphocytes was from buffy coats of 4 healthy donors (2 females, 30 and 50 years old, and 2 males, 34 and 39 years old) obtained from the Institute of Hematology and Blood Transfusion in Prague. Peripheral blood mononuclear cells (PBMCs) were isolated and cryopreserved as previously described^42,80^. The patient and healthy volunteers provided a prior signed written informed consent for the use of the biological material for future research. The research was approved according to the ethical standards of the institutional research committee—the Ethics Committee of the Motol University Hospital in Prague (approval date: Jun 19, 2019), and performed in compliance with the 1964 Helsinki declaration and its later amendments or comparable ethical standards. To prepare the reactive lymphocytes, we used our previously developed protocols^42^ and customized them for ex vivo production of both autologous and allogeneic JBT19-reactive lymphocytes. First, the cryopreserved PBMCs were reconstituted overnight (2.5 × 10^6^ cells/ml) in LM/KM medium (1:1 ratio) with IL-2 (500 IU/ml; PeproTech, Rocky Hill, NJ, USA). The composition of the KM medium was RPMI 1640 medium (Thermo Scientific) with 10% fetal bovine serum (HyClone, GE Healthcare Life Sciences, South Logan, UT, USA), 100 U/mL penicillin–streptomycin, 2 mM GlutaMax (Thermo Scientific). The reconstituted cells were then stimulated (day 0) with JBT19 cells freshly inactivated with both γ (96 Gy) and UV (312 nm, 2.55 J/cm^2^) irradiation performed as described^42,81^. The JBT19:PBMC ratio was 1:4, and the stimulation and subsequent cell culture were performed in LM/KM medium (1:1 ratio) supplemented with IL-2 (500 IU/ml). On every second or third day of cell culture, the cells were supplemented with an equal volume of fresh LM/KM medium (1:1 ratio) supplemented with IL-2 (500 IU/ml). On day 7 of culture, the cultured cells were counted and supplemented with freshly inactivated JBT19 cells at a ratio 1:4 (JBT19 : PBMC) and IL-2 (500 IU/ml). On the following second or third day of cell culture, the cells were supplemented with an equal volume of LM/KM medium (1:1 ratio) supplemented with IL-2 (500 IU/ml). On day 14 of the cell culture, the cells were analysed. As a control, the K-562 cell line (CCL-243; ATCC, Manassas, VA, USA) was used instead of JBT19 cells during the procedures. K-562 cells were maintained in KM medium.

### Analyses of autologous and allogeneic JBT19-reactive T, NK, and NKT-like cells

The cells were analyzed with small modifications using procedures described previously^42^. Briefly, the 14-day-cultured K-562- or JBT19-primed lymphocytes were harvested, pelleted by centrifugation, and resuspended in LM/KM medium (1:1 ratio) with IL-2 (250 IU/ml). The cells were then stimulated with K-562 or JBT19 cells at a ratio 1:5 (stimulant:lymphocytes). After 1 h of stimulation, the cells were supplemented with Brefeldin A (BioLegend). The cells were stimulated for 5 h and then stained with live/dead Aqua fixable stain (Thermo Scientific), fixed, permeabilized, and stained with CD3-PerCP-Cy5.5 (clone SK7), CD4-PE-Cy7 (clone MEM-241) (eBiosciences), CD8-Alexa Fluor 700 (clone MEM-31) and CD56-FITC (clone MEM-188) (Exbio), and TNFα-APC (clone MAb11), and IFNγ-PE (clone B27) (Becton Dickinson) or LAIR-1(CD305)-PE (clone NKTA255) (Thermo Scientific). The stained cells were analyzed with flow cytometry as described above. The frequency of reactive (effector) cells was calculated as the difference between the frequency of the cytokine-producing cell population of the vehicle-stimulated sample and the target cell-stimulated sample of the same donor.

### Analysis of the cytotoxic impact of JBT19-reactive lymphocytes on JBT19 cells

JBT19 cells were transfected with MISSION^®^ pLKO.1-puro-CMV-TurboGFP transduction particles (Sigma-Aldrich, cat# SHC003V) to generate TurboGFP stably expressing JBT19 cells. The transduction and antibiotic selection (0.5–1.0 µg/ml of puromycin, Sigma-Aldrich) were performed as described previously^47^. The cytotoxic assay was performed by coculture of adherent TurboGFP-JBT19 cells with the 14-day-cultured K562- or JBT19-primed lymphocytes using the procedures described previously^47^. Briefly, 0.1 × 10^6^ TurboGFP-JBT19 cells were seeded in a flat-bottom 48-well plate well (Nalgene) and cultured for 2 days. The supernatant was removed, and 0.5 × 10^6^ lymphocytes in 1 ml of LM medium with IL-2 (250 IU/ml) were added. Following the lymphocyte sedimentation for 10 min, the mean fluorescence intensity (MFI) of the TurboGFP-JBT19 cells in the wells (day 0) was determined by fluorescent microscopy and image analysis as detailed in Ref.^47^. The cells were then cocultured for 6 days. On days 1, 4, and 6 of the cell culture, the MFI of TurboGFP-JBT19 cells in the wells was determined as on day 0.

### Statistical analysis

The values were calculated from the sample size (n) using GraphPad Prism 9 (GraphPad Software, La Jolla, CA, USA). The statistical significance (*p < 0.05) between two variables was determined by paired two-tailed Student’s t-test.

### Ethical statement

All experimental protocols were approved by the ethical standards of the institutional, national research committee—the Ethics Committee of the Motol University Hospital in Prague (approval date: Jun 19, 2019). All experiments were performed in accordance with the 1964 Helsinki declaration and its later amendments or comparable ethical standards. All patients included in the study signed informed consent for the use of their blood-derived products for future research. Animal studies were approved by the Departmental Commission of the Academy of Sciences of the Czech Republic, according to Act of the Czech National Council no. 246/1992 Coll., and according to § 5 of Decree no. 419/2012 Coll., the Act on the Protection of Animals Against Cruelty under Project No. 111/2019. This study is reported according to the ARRIVE guidelines (https://arriveguidelines.org). The housing conditions and the experimental procedures respected the rules of the European Convention for the Care and Use of Laboratory Animals (2010/63/EU) as approved by the Czech Animal Care and Use Committee and by the Institutional Ethics Committee.

### Supplementary Information

Supplementary Information.

## Data Availability

All transcriptomic data are available in the ArrayExpress database under accession E-MTAB-12758, https://www.ebi.ac.uk/biostudies/arrayexpress/studies/E-MTAB-12758).
