# Supplementary material for: Novel PD-L1- and collagen-expressing patient-derived cell line of undifferentiated pleomorphic sarcoma (JBT19) as a model for cancer immunotherapy
Source: Sci Rep. 2023 Nov 4;13:19079. doi: 10.1038/s41598-023-46305-7 (PMC10625569; doi:10.1038/s41598-023-46305-7)
Supplement: Supplementary file 1 — Supplementary Information. [file 41598_2023_46305_MOESM1_ESM.pdf]

Novel PD-L1- and collagen-expressing patient-derived cell line of undifferentiated pleomorphic sarcoma (JBT19) as a model for cancer immunotherapy

Pavla Taborska[1], Pavol Lukac[4,8], Dmitry Stakheev[1,4], Lenka Rajsiglova[4,8], Katerina Kalkusova[1], Karolina Strnadova[2,3], Lukas Lacina[2,3,9], Barbora Dvorankova[2,3], Jiri Novotny[5], Michal Kolar[5], Milena Vrana[6], Hana Cechova[6], Sarka Ransdorfova[7], Marie Valerianova[7], Karel Smetana Jr[2,3], Luca Vannucci[4], and Daniel Smrz[1,4*]

[1]Department of Immunology, Second Faculty of Medicine, Charles University, and Motol University Hospital, Prague, Czech Republic

[2]Institute of Anatomy, First Faculty of Medicine, Charles University, Prague, Czech Republic

[3]BIOCEV, First Faculty of Medicine, Charles University, Vestec, Czech Republic

[4]Laboratory of Immunotherapy, Institute of Microbiology of the Czech Academy of Sciences, Prague, Czech Republic

[5]Laboratory of Genomics and Bioinformatics, Institute of Molecular Genetics of the Czech Academy of Sciences, Prague, Czech Republic

[6]HLA Department, Institute of Hematology and Blood Transfusion, Prague, Czech Republic

[7]Department of Cytogenetics, Institute of Hematology and Blood Transfusion, Prague, Czech Republic

[8]Department of Cell Biology, Faculty of Science, Charles University, Prague, Czech Republic

[9]Department of Dermatovenerology, First Faculty of Medicine, Charles University, and

General University Hospital, Prague, Czech Republic

*Address correspondence to Dr. Daniel Smrz: Department of Immunology, Second Faculty of Medicine, Charles University and Motol University Hospital Motol, V Uvalu 84, 150 06 Praha 5, Czech Republic; Phone: +420 224-43-5968; Fax: +420 224 435 962; E-mail: daniel.smrz@lfmotol.cuni.cz

Running title: Novel cell line of undifferentiated pleomorphic sarcoma

Keywords: undifferentiated pleomorphic sarcoma, PD-L1, PD-1, collagen, LAIR-1, immunotherapy

Supplemental figure legends

Figure S1. Karyotype and I-FISH analyses of JBT19 cells. (**a**) The karyotype of JBT19 cells described according to the international system for human cytogenomic nomenclature (ISCN) 2020 [1]. (**b**) The XL CDKN2A locus-specific probe detects deletions in band 9p21. This probe is labeled in orange and hybridizes to CDKN2A (p16) as well as CDKN2B (p15). The green labeled probe hybridizes to the centromere of chromosome 9 and functions as a reference probe. A representative image is shown. The deletion was detected in 174 out of 200 (87%) tested interphase nuclei.

Figure S2. **Transcription profile of the JBT19 cell line closely resembles undifferentiated pleomorphic sarcoma or myxofibrosarcoma clinical samples.** When combined in a pseudo bulk sample, the JBT19 cells display the expression profile that matches the undifferentiated pleomorphic sarcoma or myxofibrosarcoma samples from the TCGA SARC study. The heatmap displays the scaled expression intensity of the markers of iClusters identified in the original study [2]. The JBT19 cell line belongs to the iCluster 5, which represents mainly the samples of undifferentiated pleomorphic sarcoma and hardly distinguishable myxofibrosarcoma. MPNST stands for malignant peripheral nerve sheath tumor.

Figure S3. Characterization of K-562- or JBT19-primed and expanded healthy donors' and patient's NK and T cells after the stimulation (**a**) NK and T cell population frequencies of the K-562-primed and expanded healthy donors' (HD) and

patient's (Pac) lymphocytes determined using the gating strategy in Fig. 7a. (**b**) Same tests like in **a** but using JBT19-primed and expanded HD and Pac lymphocytes. Bars represent the mean of values and SEM determined in each group (HD, *n* = 4 donors; Pac, *n* = 2 preparations). White bars are resting cells (No stim), grey bars are lymphocytes stimulated with JBT19 cells (JBT-19 stim), and black bars are lymphocytes stimulated with K-562 cells (K-562 stim).

References

1 McGowan-Jordan, J., Hastings, R. J. & Moore, S. *ISCN 2020: An International System for Human Cytogenomic Nomenclature (2020)*. (Karger, Basel, 2020).

2 Cancer Genome Atlas Research Network. Electronic address, e. d. s. c. & Cancer Genome Atlas Research, N. Comprehensive and Integrated Genomic Characterization of Adult Soft Tissue Sarcomas. *Cell* **171**, 950-965 e928, doi:10.1016/j.cell.2017.10.014 (2017).

a

98~109<4n>,XX,+del(X)(?),+der(X)t(X;?)(p?;?),der(Y)t(Y;21)(p11.2;?)t(6;21)(?;?)x2,+1,+del(1)(?)x2,+del(1)(?),der(1)del(1)(p?)t(1;11)(q?;q?)x2,+del(2)(q?12)x2,der(3)del(3)(p?)del(3)(q?)x2,der(3)t(3;17)(q?21;q11.2)x2,+der(3)t(3;5)(p?12;?)t(3;17)(q?21;q11.2),der(4)del(4)(p?14)del(4)(q?13)x2,+der(4)t(4;13)(p?16;?)del(4)(q?)x2,-6,t(6;10)(p12;p12),+7,der(7)t(7;15)(p?21;?)x2,+der(7)t(7;15)(p?21;?)del(7)(q?),+der(7)del(7)(p?12)t(7;17)(q?11.2;?)t(6;17)(?;?),+8,+8,del(8)(p?)x2,+der(8)t(4;8)(?;p?11.2)x2,+9,+9,del(9)(?),der(9)t(9;19)(p?;?),-11,-11,der(11)t(11;19)(p15;?)x2,der(12)(?),-13,der(13;15)(q10;q10),-14,-14,der(14)t(7;14)(?;p?13),del(15)(q?)x2,+der(15)t(9;15)(q?;p?12),+der(15)t(10;15)(q?;p?12),+16,der(16)t(11;16)(?;q?22),der(16)t(16;?)(p11.2;?)t(11;16)(?;q?22),der(16)t(16;17)(p11.2;?)t(11;16)(?;q?22),-17,-17,del(17)(q?),del(18)(q?),der(19)del(19)(?)t(3;19)(?;?)x2,der(19)t(19;21)(?;q?)x2,+der(19)t(12;19)(q?;?)t(3;19)x2,der(20)t(17;20)(?;?)x2,ider(20)(q?10)ins(20;16)(q?12,?),der(20)t(5;20)(?;p?)ins(20;16)(q?12,?),-21,der(21)t(6;21)(?;q?)x2,+22,ider(22)(q10)t(3;22)(?;q11.2?)x2[cp16]

b

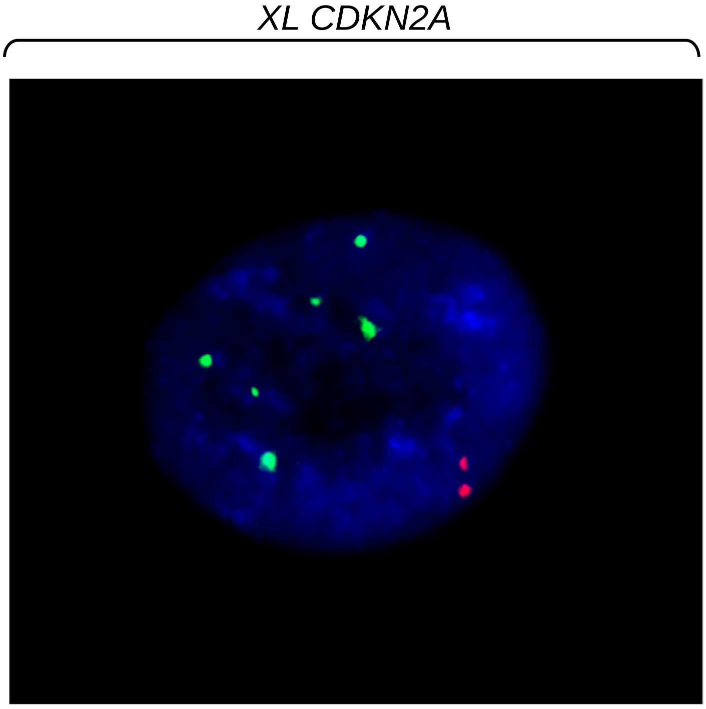

Fig. S1

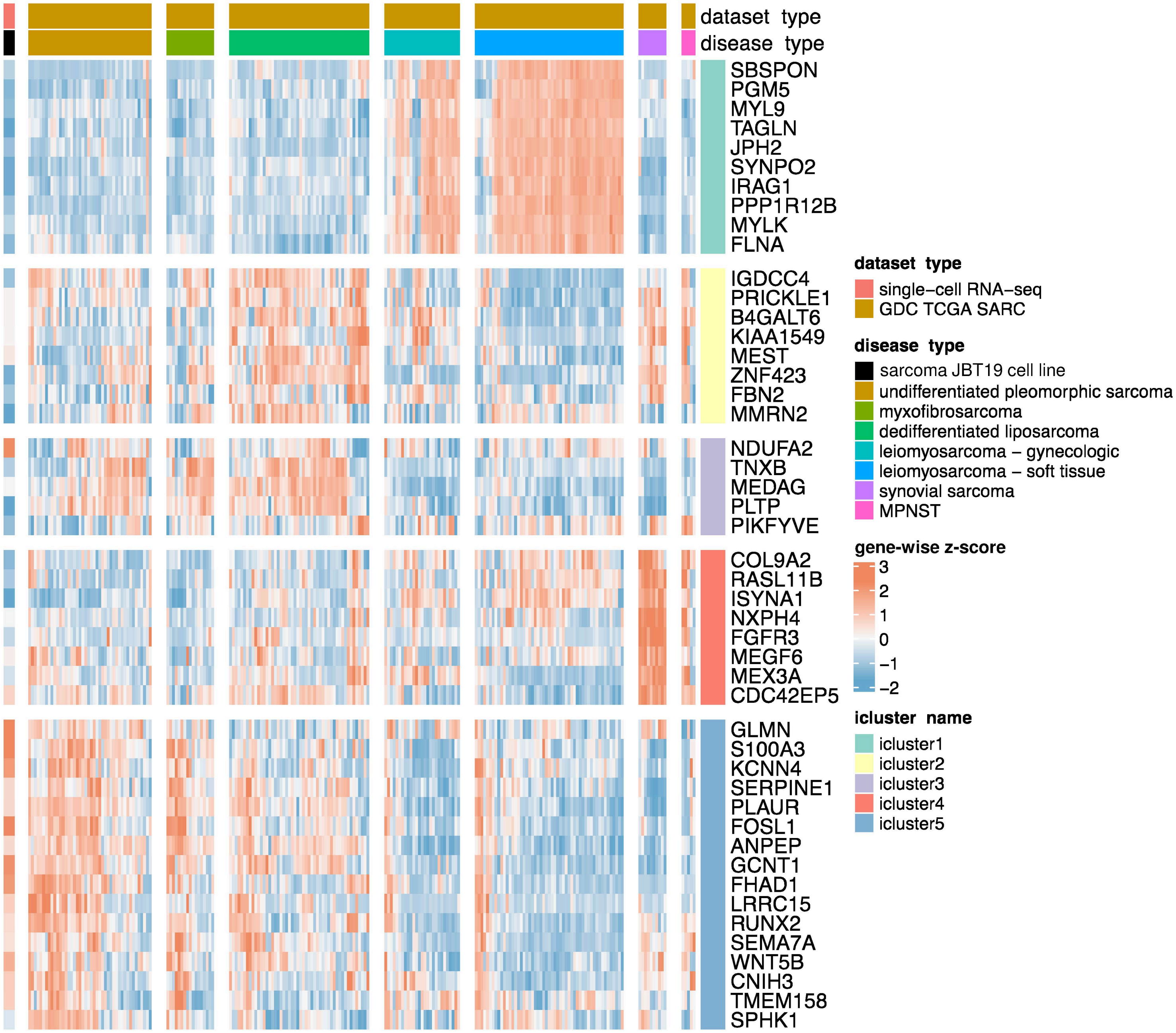
dataset type
disease type
SBSPON
PGM5
MYL9
TAGLN
JPH2
SYNPO2
IRAG1
PPP1R12B
MYLK
FLNA
IGDCC4
PRICKLE1
B4GALT6
KIAA1549
MEST
ZNF423
FBN2
MMRN2
NDUFA2
TNXB
MEDAG
PLTP
PIKFYVE
COL9A2
RASL11B
ISYNA1
NXPH4
FGFR3
MEGF6
MEX3A
CDC42EP5
GLMN
S100A3
KCNN4
SERPINE1
PLAUR
FOSL1
ANPEP
GCNT1
FHAD1
LRRC15
RUNX2
SEMA7A
WNT5B
CNIH3
TMEM158
SPHK1
dataset type
single−cell RNA−seq
GDC TCGA SARC
disease type
sarcoma JBT19 cell line
undifferentiated pleomorphic sarcoma
myxofibrosarcoma
dedifferentiated liposarcoma
leiomyosarcoma − gynecologic
leiomyosarcoma − soft tissue
synovial sarcoma
MPNST
gene-wise z-score
3
2
1
0
−1
−2
icluster name
icluster1
icluster2
icluster3
icluster4
icluster5

Fig. S2

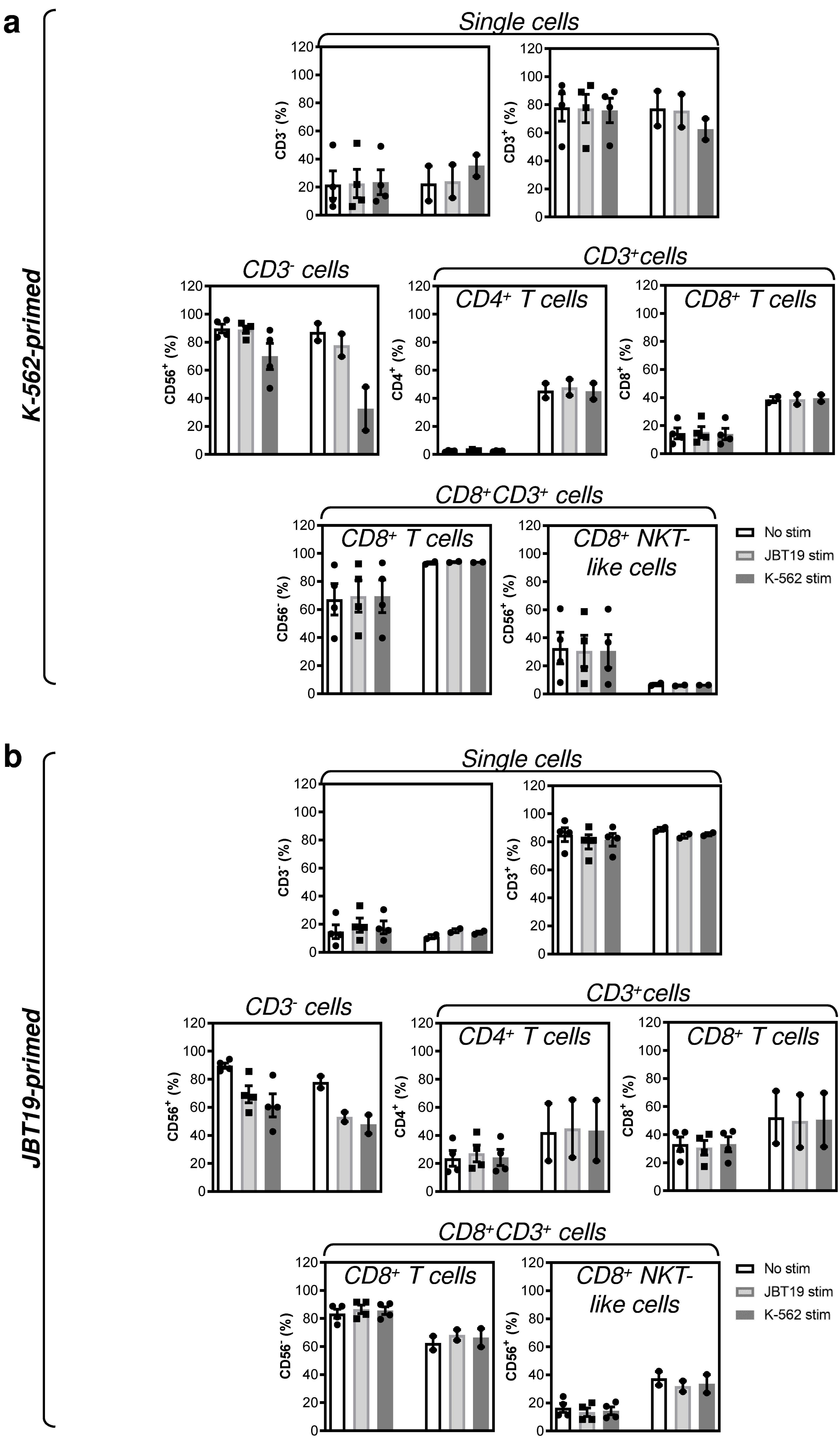

a
K-562-primed
Single cells
CD3- (%)
CD3+ (%)
CD3- cells
CD3+cells
CD4+ T cells
CD8+ T cells
CD56+ (%)
CD4+ (%)
CD8+ (%)
CD8+CD3+ cells
CD8+ T cells
CD8+ NKT-like cells
CD56- (%)
CD56+ (%)
No stim
JBT19 stim
K-562 stim
b
JBT19-primed
Single cells
CD3- (%)
CD3+ (%)
CD3- cells
CD3+cells
CD4+ T cells
CD8+ T cells
CD56+ (%)
CD4+ (%)
CD8+ (%)
CD8+CD3+ cells
CD8+ T cells
CD8+ NKT-like cells
CD56- (%)
CD56+ (%)
No stim
JBT19 stim
K-562 stim

Fig. S3
